# Importance of an Axial Ln^III^–F Bond across the Lanthanide Series and Single-Molecule Magnet Behavior in the Ce and Nd Analogues

**DOI:** 10.1021/acs.inorgchem.2c00556

**Published:** 2022-06-21

**Authors:** Emma Regincós Martí, Angelos B. Canaj, Tanu Sharma, Anna Celmina, Claire Wilson, Gopalan Rajaraman, Mark Murrie

**Affiliations:** † School of Chemistry, University of Glasgow, University Avenue, Glasgow G12 8QQ, U.K.; ‡ Department of Chemistry, Indian Institute of Technology Bombay, Powai, Mumbai, Maharashtra 400076, India

## Abstract

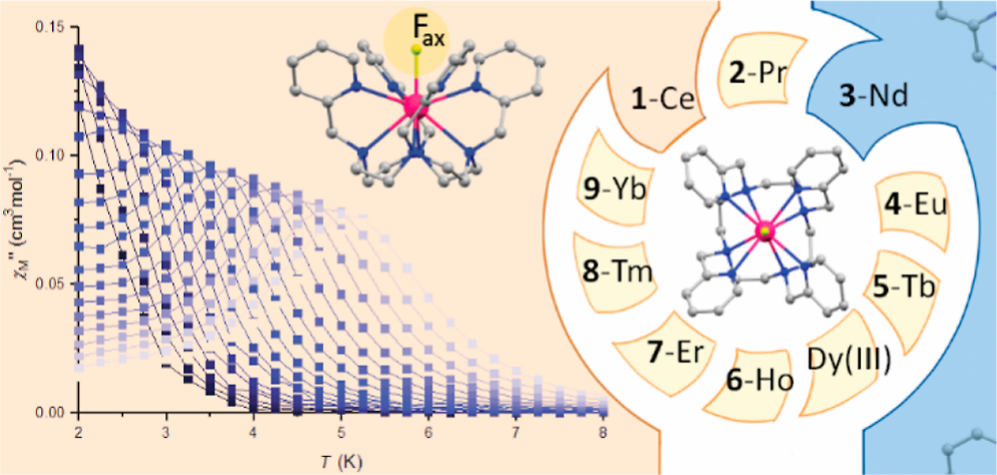

The recently reported compound [Dy^III^LF](CF_3_SO_3_)_2_·H_2_O (L = 1,4,7,10-tetrakis(2-pyridylmethyl)-1,4,7,10-tetraaza-cyclododecane) displays a strong axial magnetic anisotropy, due to the short axial Dy–F bond, and single-molecule magnet (SMM) behavior. Following our earlier [Dy^III^LF]^2+^ work, herein we report the systematic structural and magnetic study of a family of [Ln^III^LF](CF_3_SO_3_)_2_·H_2_O compounds (Ln(III) = **1**-Ce, **2**-Pr, **3**-Nd, **4**-Eu, **5**-Tb, **6**-Ho, **7**-Er, **8**-Tm, and **9**-Yb). From this series, the Ce(III) and Nd(III) analogues show slow relaxation of the magnetization under an applied direct current magnetic field, which is modeled using a Raman process. Complete active space self-consistent field theoretical calculations are employed to understand the relaxation pathways in **1**-Ce and **3**-Nd and also reveal a large tunnel splitting for **5**-Tb. Additional computational studies on model compounds where we remove the axial F^–^ ligand, or replace F^–^ with I^–^, highlight the importance of the F^–^ ligand in creating a strong axial crystal field for **1**-Ce and **3**-Nd and for promoting the SMM behavior. Importantly, this systematic study provides insight into the magnetic properties of these lighter lanthanide ions.

## Abstract 2

The structural and magnetic properties of a family of [Ln^III^LF](CF_3_SO_3_)_2_·H_2_O (L = 1,4,7,10-tetrakis(2-pyridylmethyl)-1,4,7,10-tetraaza-cyclododecane) compounds are reported. In addition to the previously reported Dy(III) analogue, we find that the Ce(III) and Nd(III) analogues show slow relaxation of the magnetization due to the strong axial magnetic anisotropy created by the axial F^−^ ligand. AC magnetic susceptibility data and CASSCF theoretical calculations are employed to understand the single-molecule magnet behavior of **1**-Ce and **3**-Nd.

## Introduction

Single-molecule magnets (SMMs) are molecular systems that have a bistable magnetic state and show magnetic hysteresis of molecular origin. They were studied for the first time in the 1990s after the magnetic characterization of [Mn_12_O_12_(MeCO_2_)_16_(H_2_O)_4_]·2CH_3_COOH·4H_2_O,^1^ also known as {Mn_12_}.^2−4^ Following studies on {Mn_12_}, the main focus of the field was the study of molecular complexes containing 3d metals, such as Mn,^5,6^ Fe,^7,8^ Ni,^9^ and Co.^10^ After 2003,^11^ the interest shifted toward lanthanide ions because of their high intrinsic magnetic moments and strong spin–orbit coupling. Both properties are important in creating a magnetic easy axis that leads to improved SMM behavior.

Some of the great advances that have been reported, such as impressive magnetization reversal barriers (*U*_eff_) and magnetic hysteresis at high temperatures,^12^ have been focused around Dy(III), followed by Tb(III)/Tb(II) and Ho(III).^13−17^ While it is understandable to focus on Dy(III) due to its Kramers ion nature, large magnetic moment (*J* = 15/2), and high magnetic anisotropy, particularly useful properties when designing high-performance SMMs,^18,19^ there is still a shortage of studies on other lanthanide ions such as Ce(III) (^2^F_5/2_) and Nd(III) (^4^I_9/2_). Recent studies into the nature of the lanthanide relaxation processes have focused on the role of Raman relaxation,^20,21^ which is often found to dominate the spin dynamics in monometallic complexes of the early lanthanides.^22,23^ Indications for this include small values of the magnetization reversal barrier^24^ that do not correspond to energy gaps between ground and excited m_J_ states and unreasonable values of the pre-exponential factor τ_0_ when including an Orbach term.^25,26^ Understanding these relaxation processes is also a vibrant research area that is relevant to all spin-based systems.^27,28^

Like Dy(III), both Ce(III) and Nd(III) have an oblate (equatorially expanded) electron distribution of the lowest *J* state. They are also both Kramers ions, which means their ground state is always doubly degenerate.^18^ This explains why one of the highest *U*_eff_ values recorded for Ce(III) and Nd(III) is for an aza-crown complex with nitrates in the axial positions.^29^ In order to promote SMM behavior for such lanthanide ions, it is useful to design a coordination environment that creates strong axial magnetic anisotropy. If two suitable axial ligands are provided, such as in the case of pseudo-*D*_5*h*_ complexes of Dy(III),^30^ one can obtain even zero-field single-ion magnets (SIMs) of Ce(III) and Nd(III), albeit with smaller energy barriers.^31,32^ Herein, we study a coordination environment that uses a fluoride ligand in the axial position to create a strong axial crystal field, following previous positive results with Dy(III) by us, using an octadentate N_8_ ligand and Norel *et al.*, using a combination of a hexadentate N_6_ ligand with either two pyridine or 1,4-dioxane ligands.^33,34^

The ligand 1,4,7,10-tetrakis(2-pyridylmethyl)-1,4,7,10-tetraaza-cyclododecane, L, is a cyclen with four flexible “arms” containing pyridine and can encapsulate a metal through its eight nitrogen atoms (see Figure S1). The ligand L has been studied for its coordination with different metal ions,^35−41^ including lanthanides.^42−47^ With 3d metals, the ligand forms a pocket that fully encapsulates the metal. On the other hand, with lanthanides being larger cations, there is enough space in the first coordination sphere for another smaller ligand. This theory was tested by our group with the study of [Dy^III^LF](CF_3_SO_3_)_2_·H_2_O, which behaves as an SMM.^33^ We showed that the fluoride ligand, due to its small size and negative charge, is an ideal candidate to create a strong axial magnetic anisotropy and generate the highest magnetization reversal barrier among a group of monodentate ligands (*i.e.*, OH^–^, HCO_2_^–^, ^*t*^BuCO_2_^–^, CF_3_CO_2_^–^, CH_3_CH_2_CH_2_O^–^, and ^*t*^BuCH_2_CH_2_O^–^) that were tested in silico.^33^

Therefore, we decided to synthesize and study other analogues of this system with a terminal Ln(III)–F bond. Our synthetic strategy led to compounds with the formula [LnLF](CF_3_SO_3_)_2_·H_2_O: Ln(III) = Ce (**1-**Ce), Pr (**2-**Pr), Nd (**3-**Nd), Eu (**4-**Eu), Tb (**5-**Tb), Ho (**6-**Ho), Er (**7-**Er), Tm (**8-**Tm), and Yb (**9-**Yb) plus diluted compounds **10-**Ce@La, **11-**Nd@La, and **12-**Tb@Y. We refer the interested reader to our earlier publication for full details on [Dy^III^LF](CF_3_SO_3_)_2_·H_2_O.^33^ Importantly, **1-**Ce and **3-**Nd show slow relaxation of the magnetization under an applied direct current (dc) field, expanding the limited family of reported Ce(III) and Nd(III) SMMs.

## Experimental Methods

All reagents were used as received without further purification. No safety hazards were encountered during the described experimental procedures.

1,4,7,10-Tetrakis(2-pyridylmethyl)-1,4,7,10-tetraaza-cyclododecane (L): 2-(chloromethyl) pyridine hydrochloride (2.51 g, 15.3 mmol) and cyclen (0.65 g, 3.87 mmol) were refluxed overnight in 60 mL of MeCN and excess Cs_2_CO_3_ (24.9 g, 76.4 mmol). The reflux gave a dark red solution that was filtered in vacuo and washed multiple times with CH_2_Cl_2_. Rotary evaporation of the filtrate gave **L** in the form of yellow crystals (yield = 1.60 g, 78%). ^1^H NMR in CDCl_3_ at 298 K: δ 2.79 (16 H, s, NC***H***_***2***_C***H***_***2***_N), 3.66 (8 H, s, NC***H***_***2***_(C_5_H_4_N)), 7.11 (4 H, ddd, *J* = 12.4 Hz, *J* = 7.8 Hz, *J* = 1.2 Hz, C_5_***H***_***4***_N), 7.44 (4 H, td, *J* = 7.8 Hz, *J* = 1.2 Hz, C_5_***H***_***4***_N), 7.74 (4 H, d, *J* = 7.8 Hz, C_5_***H***_***4***_N), 8.50 (4 H, dd, *J* = 4.8 Hz, *J* = 1.2 Hz, C_5_***H***_***4***_N), 1.67 (H_2_O, s, solvent) 7.29 (CHCl_3_, s, solvent) (see Figure S2).

For the synthesis of complexes **1**-Ce to **12**-Tb@Y, the following procedure is applicable: Ln(CF_3_SO_3_)_3_ (0.08 mmol) was dissolved in 4 mL of MeOH along with L (47 mg, 0.08 mmol) and refluxed overnight. The solution was then dried in vacuo until an oil resulted, which was redissolved in 10 mL of MeCN and refluxed for a further 2 h. After drying the solution in vacuo again, the obtained oil was dissolved in 4 mL of CH_3_Cl, stirred at high temperature, and dried in vacuo to obtain a white solid. The solid was dissolved in water with NH_4_F (yield depending, 4 equiv) and stirred at high temperature for 10 min. After filtration and slow evaporation, colorless crystals formed in 2–3 days. For the 10% diluted analogues, **10**-Ce@La to **12**-Tb@Y, the same procedure is applicable by using a combination of salts, 0.072 mmol La(CF_3_SO_3_)_3_ or Y(CF_3_SO_3_)_3_ and 0.008 mmol Ln(CF_3_SO_3_)_3_ (Ln(III) = Ce, Nd, and Tb) in the first step.

**1**-Ce [Ce(L)F](CF_3_SO_3_)_2_·H_2_O yield 53 mg. Elemental Anal. Calcd (found): C, 40.34 (40.30); H, 4.19 (4.05); N, 11.07 (10.94)%.

**2**-Pr [Pr(L)F](CF_3_SO_3_)_2_·H_2_O yield 49 mg. Elemental Anal. Calcd (found): C, 40.32 (40.43); H, 4.19 (4.15); N, 11.06 (11.03)%.

**3**-Nd [Nd(L)F](CF_3_SO_3_)_2_·H_2_O yield 31 mg (56.3%). Elemental Anal. Calcd (found): C, 40.19 (40.18); H, 4.17 (4.08); N, 11.03 (10.94)%.

**4**-Eu [Eu(L)F](CF_3_SO_3_)_2_·H_2_O yield 24 mg (44.4%). Elemental Anal. Calcd (found): C, 39.89 (39.90); H, 4.13 (4.03); N, 10.94 (10.88)%.

**5**-Tb [Tb(L)F](CF_3_SO_3_)_2_·H_2_O yield 14 mg (25.4%). Elemental Anal. Calcd (found): C, 39.62 (39.56); H, 4.11 (4.09); N, 10.87 (10.72)%.

**6**-Ho [Ho(L)F](CF_3_SO_3_)_2_·H_2_O yield 40 mg (30.5%). Elemental Anal. Calcd (found): C, 39.39 (39.61); H, 4.08 (4.07); N, 10.81 (10.89)%.

**7**-Er [Er(L)F](CF_3_SO_3_)_2_·H_2_O yield 20 mg (35.4%). Elemental Anal. Calcd (found): C, 39.30 (39.45); H, 4.07 (4.05); N, 10.78 (10.87)%.

**8**-Tm [Tm(L)F](CF_3_SO_3_)_2_·H_2_O yield 30 mg (22.7%). Elemental Anal. Calcd (found): C, 39.24 (39.48); H, 4.07 (4.02); N, 10.77 (10.9)%.

**9**-Yb [Yb(L)F](CF_3_SO_3_)_2_·H_2_O yield 7 mg (12.7%). Elemental Anal. Calcd (found): C, 39.08 (38.92); H, 4.05 (3.96); N, 10.72 (10.55)%.

**10**-Ce@La [La_0.9_Ce_0.1_(L)F](CF_3_SO_3_)_2_·H_2_O. Elemental Anal. Calcd (found): C, 41.14 (40.91); H, 4.06 (3.95); N, 11.29 (11.03)%.

**11**-Nd@La [La_0.9_Nd_0.1_(L)F](CF_3_SO_3_)_2_·0.5H_2_O. Elemental Anal. Calcd (found): C, 40.77 (41.1); H, 4.13 (4.01); N, 11.19 (10.89)%.

**12**-Tb@Y [Y_0.9_Tb_0.1_(L)F](CF_3_SO_3_)_2_·0.5H_2_O. Elemental Anal. Calcd (found): C, 43.11 (43.05); H, 4.31 (4.25); N, 11.83 (11.97)%.

## Theoretical Calculations

In order to rationalize the magnetic properties observed through experiments, *ab initio* calculations were performed using the MOLCAS 8.2 suite.^48−50^ We used [ANO-RCC···8s7p5d3f2g1h]^51^ for Ce, Nd, and Tb atoms; [ANO-RCC···3s2p1d] for C, N, and F atoms; and [ANO-RCC···2s1p] for H atoms. Ce(III), Nd(III), and Tb(III) (f^1^, f^3^, and f^8^) have a ^2^F_5/2_, ^4^I_9/2_, or ^7^F_6_ ground state, respectively. Complete active space self-consistent field (CASSCF) calculations were carried out considering one electron in seven active orbitals [CAS(1,7)] in **1-**Ce, three electrons in seven active orbitals [CAS(3,7)] in **3-**Nd, and eight electrons in seven active orbitals [CAS(1,7)] in **5-**Tb. Further full CI method was employed to compute 7 doublets in **1-**Ce, 35 quartets and 112 doublets in **3-**Nd, and 7 heptets, 140 quintets, and 588 triplets in **5**-Tb. All of these computed spin states are spin-free states. Afterward, using the restrictive active space spin-state interaction spin–orbit (RASSI-SO) program,^52^ 7 doublets were mixed in **1-**Ce, 35 quartets and 112 doublets in **3-**Nd, and 7 septets, 140 quintets, and 195 triplets in **5-**Tb. Furthermore, these computed SO states were taken in the SINGLE_ANISO code,^49^ and g-tensors and other local magnetic properties were obtained. The model complexes were optimized using DFT calculations, employing the UB3LYP functional^53,54^ along with SDD^55,56^ for Y and 6-31G* for the other atoms, employing the G09 suite of programs.^57^

## Results and Discussion

### Description of the Crystal Structures

Single crystals were obtained for **1**-Ce, **2**-Pr, **3**-Nd, **4**-Eu, **5**-Tb, **6**-Ho, **7**-Er, **8**-Tm, **9**-Yb, **10**-Ce@La, **11**-Nd@Y, and **12**-Tb@Y, all of them are columnar prisms that gave good-quality single-crystal data (see Tables S1–S6). The crystal system is orthorhombic for all analogues; however, the space group depends on the size of the lanthanide ion. The larger lanthanides (**1**-Ce to **3-**Nd**, 10**-Ce@La, and **11**-Nd@La) crystallize in the centrosymmetric *Pccn* group, where the complexes are related by inversion, while the smaller ones (**4**-Eu to **9**-Yb and **12**-Tb@Y) crystallize in the enantiomorphic *P*2_1_2_1_2 group. The diluted samples were prepared with either La(III) or Y(III) to be consistent with the space group: La(III) crystallizes in the *Pccn* group, as do **1**-Ce and **3**-Nd, whereas Y(III) crystallizes in *P*2_1_2_1_2 as does **5**-Tb.

In all cases, the asymmetric unit comprises a half-ligand L (which is completed by two-fold rotation) surrounding the metal, a fluoride ligand, the counterion CF_3_SO_3_^–^, and a co-crystallized water molecule; the metal and fluoride and the co-crystallized water oxygen atom all lie on a 2-fold rotation axis with only 0.5 of each of these atoms in the asymmetric unit. All analogues pack in compact columns with the Ln(III)···Ln(III) intermolecular distances ranging from 7.95(9) Å (for **1**-Ce) to 7.76(8) Å (for **9**-Yb). Representative examples of the crystal packing in the *Pccn* and *P*2_1_2_1_2 groups can be found in Figure S3.

**Figure 1:**
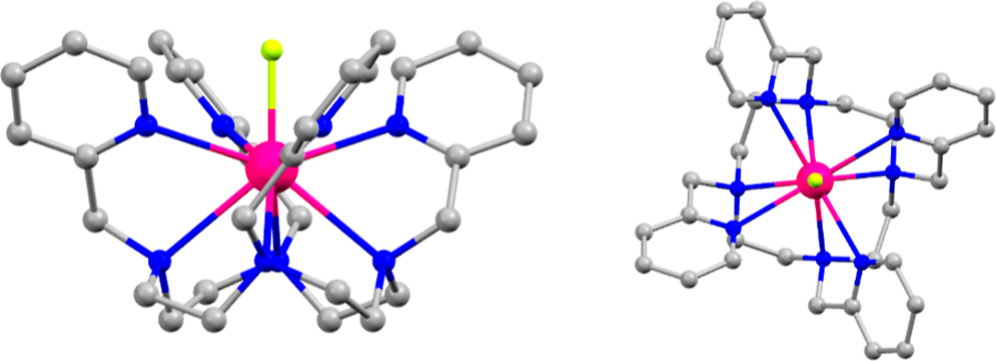
Structure of the cationic complex [Nd^III^LF]^2+^ in **3**-Nd viewed along the *a*-axis (left) and *c*-axis (right). C, gray; N, blue; Nd, pink; F, lime green; and H omitted for clarity.

The lanthanide ion is coordinated with four nitrogen atoms of the aza-crown and encapsulated by four nitrogen atoms belonging to the pyridine group of the flexible arms of the ligand (see Figure 1). The Ln–N bond distances are longer for the nitrogen atoms belonging to the aza-crown than for the nitrogen atoms belonging to the pyridine groups, indicating that the lanthanide ion is not placed equidistantly within the [N_8_] cage, leaving space for further coordination with the electronegative anionic fluoride ligand. The Ln–F bond distance increases with the increasing ionic radius of the lanthanide ions, from 2.096(3) Å for **9-**Yb to 2.206(3) Å for **1**-Ce (see Tables 1 and S4–S6 for details). This distance is considerably shorter than the distances for the Ln-N bonds, which range from 2.507(5) to 2.748(3) Å (see Tables 1 and S4–S6 for details). This [N_8_F] coordination environment is particularly promising to generate an axial crystal field for lanthanide ions with an oblate 4f-electron distribution, which is the case for the Kramers ions Ce(III), Nd(III), and Dy(III) and the non-Kramers ions Pr(III), Tb(III), and Ho(III).^18,58^

**Table 1:** Selected Bond Lengths and SHAPE Studies for the [Ln^III^LF]^2+^ Complexes

	space group	Pseudo-symmetry	SHAPE studies	Ln–F bond length (Å)	Ln–N_py_ avg. bond length (Å)	Ln–N_crown_ avg. bond length (Å)	Ln···Ln closest distance (Å)
**1**-Ce	*Pccn*	capped square antiprism (*C*_4*v*_)	2.51	2.21	2.68	2.76	7.96
**2**-Pr	*Pccn*		2.42	2.19	2.66	2.75	7.96
**3**-Nd	*Pccn*		2.40	2.19	2.64	2.73	7.96
**4**-Eu	*P*2_1_2_1_2		0.61	2.16	2.57	2.7	7.77
**5**-Tb	*P*2_1_2_1_2		0.57	2.14	2.55	2.69	7.76
Dy(III)^33^	*P*2_1_2_1_2		0.58	2.12	2.53	2.68	7.76
**6**-Ho	*P*2_1_2_1_2		0.51	2.13	2.52	2.67	7.76
**7**-Er	*P*2_1_2_1_2		0.54	2.13	2.52	2.67	7.76
**8**-Tm	*P*2_1_2_1_2		0.58	2.11	2.50	2.67	7.76
**9**-Yb	*P*2_1_2_1_2		0.53	2.10	2.50	2.66	7.77

**Figure 2:**
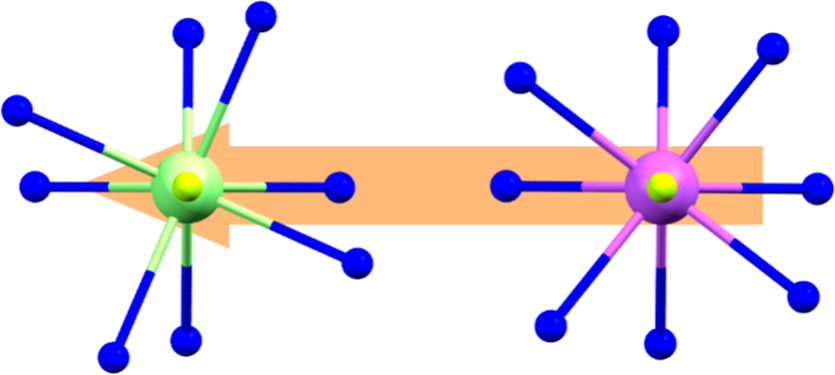
First coordination sphere of the lanthanide ions showing the increase in distortion from **9**-Yb (pink) to **1**-Ce (lime green).

All complexes were analyzed using SHAPE,^59−61^ which compares the atomic coordinates to those of an ideal prism, with a value of 0 indicating a perfect match and higher values indicating higher distortion from the ideal geometry.^59^ For all complexes, the nine-coordinate environment around the lanthanide ion can be best described as a distorted capped square antiprism (see Figure S4 and Table S7), corresponding to a distorted *C*_4*v*_ symmetry, which has not been reported for Ce(III) or Nd(III) SMMs before. The distortion is greater for the larger lanthanides, and it diminishes as the size of the metal ion decreases (Figure 2). For the larger lanthanide ions (**1**-Ce, **2**-Pr, and **3**-Nd, see Table 1) which crystallize in the *Pccn* space group, the SHAPE values vary from 2.506 to 2.397. On the other hand, the analogues that crystallize in the *P*2_1_2_1_2 group (**4**-Eu, **5**-Tb, **6**-Ho, **7**-Er, **8**-Tm, and **9**-Yb) have values far lower, ranging from 0.605 to 0.532 (see Table 1).

Phase purity was confirmed by powder X-ray diffraction (PXRD). Within the two different space groups, *Pccn* (see Figure S5) or *P*2_1_2_1_2 (see Figure S6), the different analogues have the same powder X-ray diffraction pattern.

### Magnetic Properties

**Figure 3:**
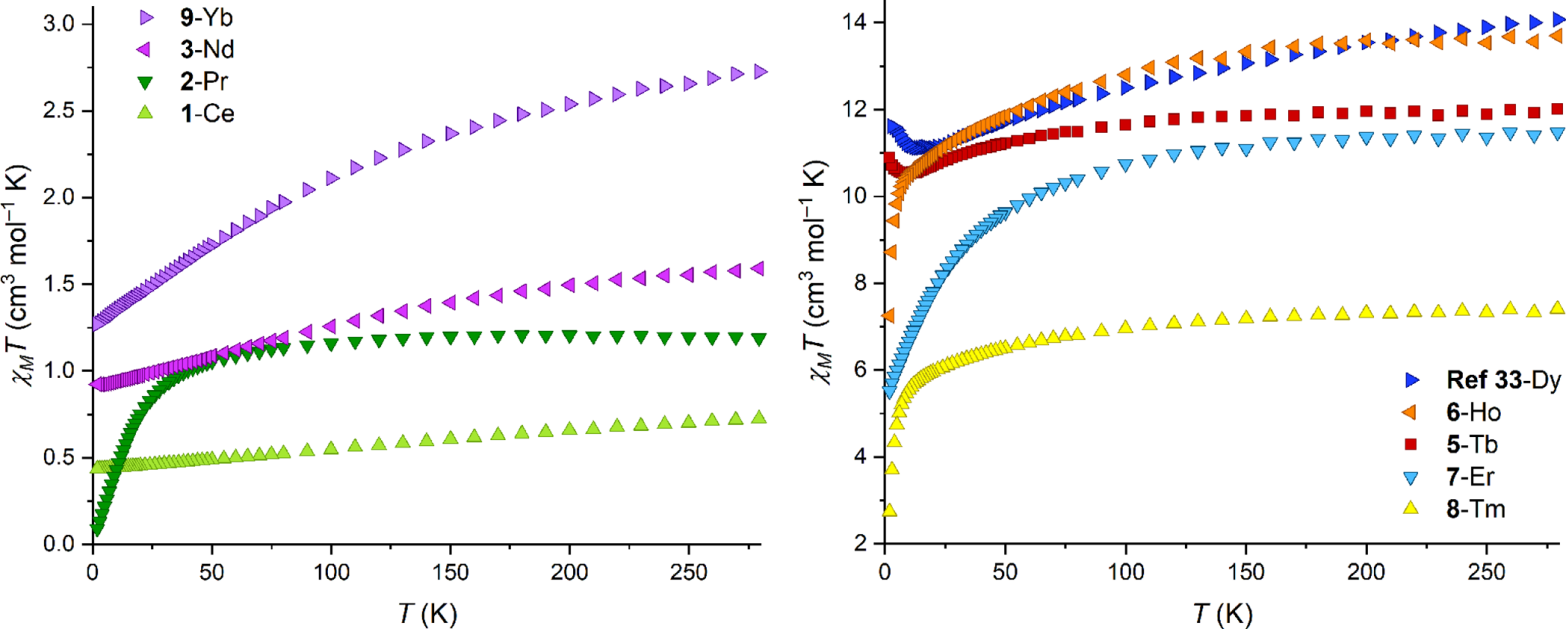
Temperature dependence of χ_M_*T* from 290 to 2 K for all analogues measured, including the previously reported Dy analogue to allow comparison of the low-temperature region with **5**-Tb.^33^

Magnetic susceptibility measurements in a dc field of 1000 Oe were recorded from 290 to 2 K for all complexes (Figure 3) except **4**-Eu (where *J* = 0). The χ_M_*T* product of all compounds is constant at higher temperatures, and then χ_M_*T* decreases upon cooling due to the depopulation of the Stark levels (Figure 3). **5-**Tb experiences an increase in χ_M_*T* from 10 K down to 2 K. The same behavior was seen for the Dy(III) analogue^33^ of this family, and it was attributed to intermolecular interactions due to the short Dy···Dy distance, 7.7 Å.^33^ The Tb···Tb distance in **5-**Tb is 7.76 Å, very similar to the case with Dy. In order to reduce the intermolecular interactions, the diluted analogue containing Y(III) and Tb(III) in a 9:1 ratio, **12**-Tb@Y, was synthesized. For this diluted sample, the χ_M_*T* product is constant at higher temperatures and then decreases, consistent with a lack of intermolecular ferromagnetic interactions (see Figure S7). The higher magnetic moments of Dy(III) and Tb(III) over other lanthanides allow them to interact at shorter distances and could explain why ferromagnetic intermolecular interactions are only seen with these two ions in the series.^62^

The χ_M_*T* products at 290 K are in agreement with the theoretical values (see Table 2 and Figure 3). We note that **1**-Ce, **2**-Pr, **6-**Ho, and **8**-Tm have experimental values lower than the theoretical ones; this has been studied previously and could be related to ligand field effects.^63,64^ The temperature dependence of χ_M_*T* was also calculated for **1**-Ce, **3**-Nd, and **5**-Tb, which can be seen in Figure S8. Magnetization experiments were performed on all analogues with a variable dc field up to 7 T (Figure S9). Saturation was not reached for any of the analogues at 2 K, with the observed magnetization values being lower than the theoretical ones (*M*_sat_), which is common when studying magnetically anisotropic complexes.^65,66^

**Table 2:** Theoretical and Experimental Values of the χ_M_*T* Product (Given in cm^3^ mol^–1^ K) and the Magnetization (Given in Nβ)

	**1**-Ce	**2**-Pr	**3**-Nd	**5**-Tb	**6**-Ho	**7**-Er	**8**-Tm	**9**-Yb
χ_M_*T*_theo_	0.80	1.60	1.64	11.81	14.06	11.48	7.15	2.57
χ_M_*T*_exp_	0.72	1.20	1.59	12.01	13.70	11.47	6.97	2.67
*M*_sattheo_	2.14	3.20	3.27	9.00	10.00	9.00	7.00	4.00
*M*_exp_	1.04	0.51	1.46	5.13	4.92	5.00	3.62	1.94

Under zero external dc field, no out-of-phase alternating current (ac) susceptibility signals were observed in any of the studied analogues, indicating no slow relaxation of the magnetization. However, when applying an optimum external dc field (Figure S10) to suppress quantum tunneling of the magnetization (QTM), **1**-Ce and **3**-Nd displayed out-of-phase ac susceptibility signals with fully formed peaks up to 5 K (see Figures S11–S14, 4, and 5).

**Figure 4:**
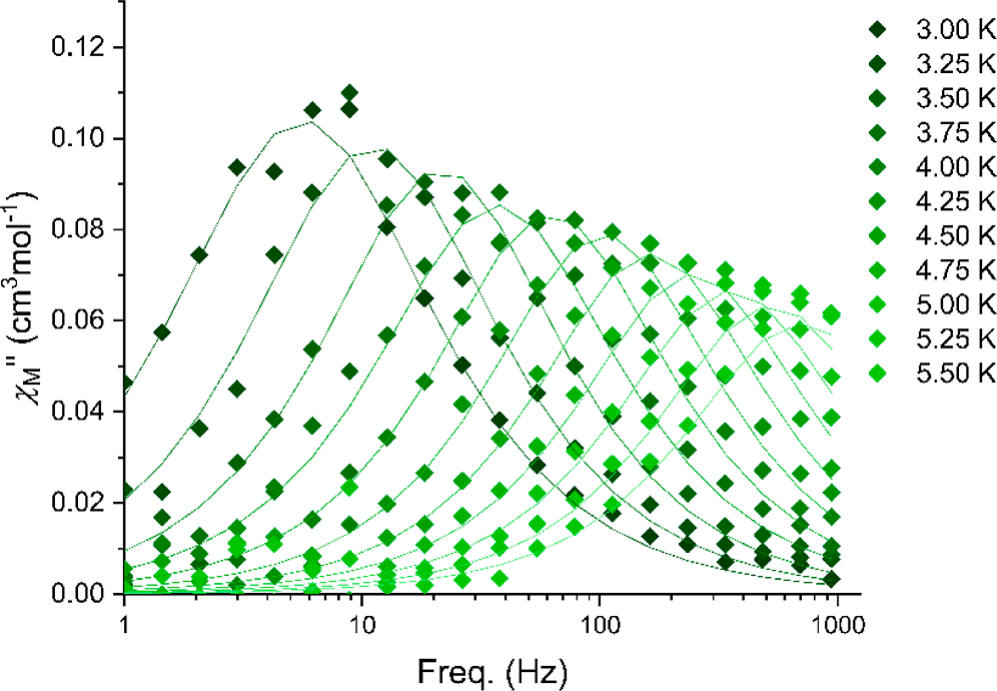
Out-of-phase ac magnetic susceptibility in an applied field *H*_dc_ = 1200 Oe for **1**-Ce.

**Figure 5:**
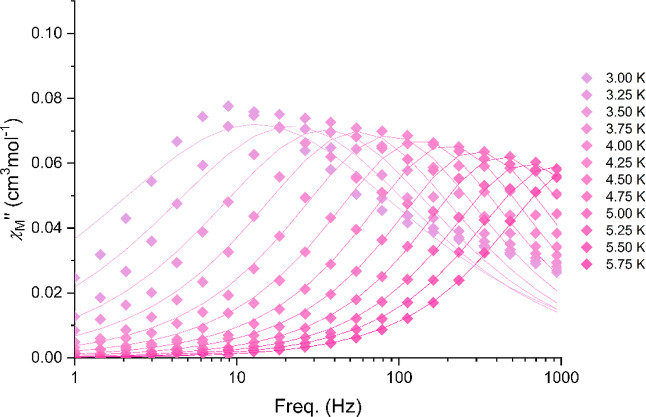
Out-of-phase ac magnetic susceptibility in an applied field *H*_dc_ = 800 Oe for **3**-Nd.

The effect of the applied dc field on the relaxation time was studied between 0 and 4000 Oe (see Figure S10). The optimum dc field that allows for the suppression of QTM was determined to be 1200 and 800 Oe for **1**-Ce and **3**-Nd, respectively.

For both **1**-Ce and **3**-Nd, it was possible to extract relaxation times from the Cole–Cole plots (see Figures S15 and S16).^67^ The relaxation times for SMMs can be fitted using different relaxation pathways by using the following equation
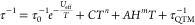
1

Equation 1 includes contributions from Orbach, where τ_0_ is the pre-exponential factor, *U*_eff_ is the magnetization reversal barrier, and *T* is the absolute temperature; Raman, where *C* is a constant and *n* has values up to 9 for lanthanides; direct, where *A* is a constant, *H* is the magnetic field, and *m* is equal to 4 for Kramers ions and 2 for non-Kramers ions; and QTM, with τ_QTM_^–1^ being the temperature-independent QTM parameter. Fitting the obtained relaxation data for **1**-Ce and **3**-Nd using eq 1 was unsuccessful. This could be due to the small temperature window in which the slow relaxation is observed, which makes separation of the different relaxation processes more difficult, and/or overparameterization. Considering this, the following simplifications were made. Due to the application of an external dc field during the ac measurements, QTM is considered to be suppressed, and therefore, the τ_QTM_^–1^ term was not taken into account for both **1**-Ce and **3**-Nd.

First, we attempted to fit the field dependence of the relaxation times (see Figure S10) by using the equation
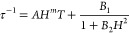
2

**Figure 6:**
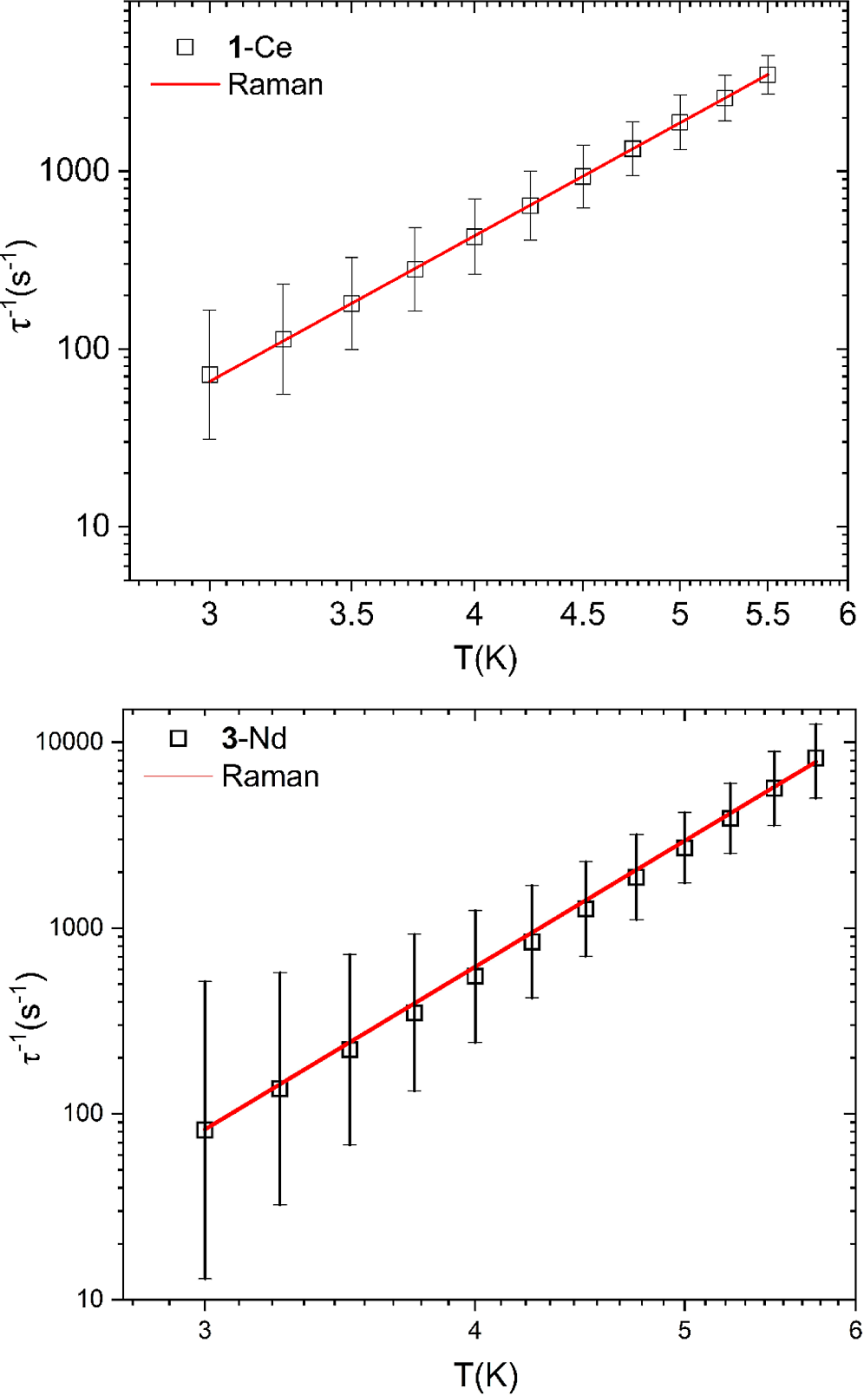
Temperature dependence of 1/τ for **1**-Ce (upper) and **3**-Nd (lower). Solid red lines represent fits for Raman relaxation τ^–1^ = *CT*^*n*^ (see text for details). Black vertical bars are estimated standard deviations in the relaxation times derived from Debye fits according to ref (67).

The direct terms are as defined above, and the second term represents the field dependence of QTM. Unfortunately, no satisfactory fit was obtained using this equation. We also tried to obtain the A parameter from fits that included the Orbach, Raman, and direct terms in eq 1: the A values obtained were negligible, and therefore, the direct relaxation process was not considered further. Next, attempts to fit the relaxation times by considering only the Orbach term and the Raman term in eq 1 yielded small values of *U*_eff_ of 37.5 and 73.7 K, for **1**-Ce and **3**-Nd, respectively, that are not consistent with the energies of the *m*_J_ states obtained from the computational studies (vide infra). However, Raman relaxation is commonly observed in Ce(III) and Nd(III) SMMs, and we were able to fit the data using only a Raman process (see Figure 6).^22,23^ The best fits’ values obtained were *C* = 0.049(1) K^–*n*^ s^–1^, *n* = 6.56(1) for **1**-Ce and *C* = 0.038(15) K^–*n*^ s^–1^, *n* = 7.0(2) for **3**-Nd, with fitting errors shown in parentheses. These values are in line with those observed previously for Ce(III) and Nd(III) SMMs.^26,29^

In order to study the magnetism of these complexes further, the diluted analogues containing La(III), **10**-Ce@La, and **11**-Nd@La were synthesized. The dilution did not have a significant effect on the magnetic properties, with the out-of-phase peaks appearing at the same temperatures as in the non-diluted analogues (see Figure S17). In addition, the diluted analogue of Tb(III) with Y(III), **12**-Tb@Y, was also studied to see if it had improved magnetic properties since **5**-Tb showed no slow relaxation of the magnetization. However, only a negligible out-of-phase ac signal was observed under an applied dc field (see Figure S18).

For Ce(III) and Nd(III) single-ion magnets, the predominant magnetic relaxation process is usually Raman relaxation, but where the Orbach process is included in the analysis, magnetization reversal barriers up to 73 K for Nd(III) and 45 K for Ce(III) are reported.^29,68^Tables 3 and 4 show a list of the single-ion magnets reported with Ce(III) and Nd(III). For both of these, the most common pseudo-symmetry falls within a dihedral group (*D*_*nh*_) in contrast to **1**-Ce and **3**-Nd that are, to the best of our knowledge, the first Ce(III)/Nd(III) SMMs reported with *C*_4*v*_ pseudo-symmetry.

**Table 3:** Ce(III) Monometallic SMMs

Ce complexes^a^	Pseudo-symmetry	*H*_dc_ (Oe)	*U*_eff_ (K)	τ_0_ (s^–1^)	*C* (K^–*n*^ s^–1^)	*n*	ref
[Ce(COT″)_2_][Li(DME)_3_]	sandwich complex	400	30	1.20 × 10^–6^			(69)
[CeCd_3_(Hquinha)_3_(*n*-Bu_3_PO)_2_I_3_]_3_EtOH_2_(H_2_O)	*D*_6*h*_	1500	27	8.20 × 10^–7^			(25)
[Ce(NO_3_)_3_(18-crown-6)]		1000	31.4	1.71 × 10^–7^			(29)
			30.3	2.20 × 10^–7^	0.1	5	
			25.6	9.00 × 10^–7^	0.0016	9	
[Ce(NO_3_)_3_(1,10-diaza-18-crown-6)]		1000	44	2.30 × 10^–8^			(29)
			45	2.60 × 10^–8^	0.52	5	
			23	6.00 × 10^–6^	0.0022	9	
Ce(fdh)_3_(bpy)		2000	33.3	1.80 × 10^–7^	0.4	6	(70)
[LCe(NO_3_)_3_]	*C*_*s*_	200			1.44	6.8	(32)
[Ce(Cp^ttt^)_2_{(C_6_F_5_-κ^1^-*F*)B(C_6_F_5_)_3_}]	sandwich complex	1000			0.0308	5.4	(23)
[Ce(Cp^ttt^)_2_(Cl)]	sandwich complex	1000			0.00475	6.5	(23)
[Ce(18-crown-6) (Cl_4_Cat) (NO_3_)]	*D*_6*h*_	1500			1.22	5 (fixed)	(22)
[Ce(18-crown-6) (Br_4_Cat) (NO_3_)]	*D*_6*h*_	800			1.87	5 (fixed)	(22)

a COT″ = bis(trimethylsilyl)cyclooctatetraenyl dianion); DME = dimethyl ether; H_2_quinha = quinaldic hydroxamic acid; fdh = 1,1,1-fluoro-5,5-dimethylhexa-2,4-dione; bpy = 2,2′-bipyridine; Cp^ttt^ = C_5_H_2_^*t*^Bu_3_-1,2,4; X_4_Cat = tetrahalocatecholate; and L = ^t^BuPO(NH^i^Pr)_2_.

**Table 4:** Nd(III) Monometallic SMMs

Nd complexes^a^	Pseudo-symmetry	*H*_dc_ (Oe)	*U*_eff_ (K)	τ_0_ (s^–1^)	*C* (K^–*n*^ s^–1^)	*n*	ref
NdTp_3_	*D*_3*h*_	100	4	4.20 × 10^–5^			(71)
[Nd(W_5_O_18_)_2_]^9–^	*D*_4*h*_	1000	73.9	3.55 × 10^–10^			(68)
[L_1_Nd(Η_2_Ο)_5_][Ι]_3_L_1_(Η_2_Ο)	*D*_5*h*_	0	16.1	2.64 × 10^–4^			(31)
	*D*_5*h*_		24.7	5.03 × 10^–6^			(31)
	*D*_5*h*_	2000	39.2	8.98 × 10^–7^			(31)
[L_1_Nd(Η_2_Ο)_5_][Ι]_3_L_1_(Η_2_Ο)	*D*_5*h*_	2000				6.3	(26)
{[Nd((μ_2_L_2_)_3_(Η_2_Ο)_2_]·C_2_H_3_Ν}_*n*_	*C*_2*v*_ CHAIN	2000	27	4.10 × 10^–7^			(65)
[Nd(μ_2_-L_3_) (L_3_) (CH_3_COO) (H_2_O)_2_]_*n*_	*D*_3*h*_ CHAIN	3500	29	3.10 × 10^–7^			(65)
[NdCd_3_(Hquinha)_3_(*n*-Bu_3_PO)_2_I_3_]·3EtOH·2H_2_O	*D*_6*h*_	2500	22	3.90 × 10^–7^			(25)
[Nd(NO_3_)_3_(18-crown-6)]		1000	29.9	2.90 × 10^–9^			(29)
			30.9	2.20 × 10^–8^	4.1	5 (fixed)	
			33.4	1.69 × 10^–9^	0.000025	9 (fixed)	
[Nd(NO_3_)_3_(1,10-diaza-18-crown-6)]		1000	69	2.10 × 10^–10^			(29)
			55	2.60 × 10^–9^	0.05	5 (fixed)	
			73	1.40 × 10^–10^	0.00107	9 (fixed)	
[Nd(CyPh_2_PO)_2_(H_2_O)_5_]I3·2(CyPh_2_PO)·3EtOH	*D*_5*h*_	0				5.1	(26)
		2000				6.5	(26)
(NH_2_Me_2_)_3_{[Nd(Mo_4_O_13_)(DMF)_4_]_3_(BTC)_2_}·8DMF	*D*_3*h*_	500	26.7	1.41 × 10^–7^			(72)
first: only Orbach, second: Raman and Orbach		500	34.1	4.69 × 10^–8^			
Nd(fdh)_3_(bpy)		500	28.8	9.20 × 10^–8^	0.93	6.6	(70)
[Nd(Cp^ttt^)_2_{(C_6_F_5_-κ^1^-*F*)B(C_6_F_5_)_3_}]	sandwich complex	1000			0.00117	6.3	(23)
[Nd(Cp^ttt^)_2_(Cl)]	sandwich complex	1000	73.6	9.64 × 10^–8^	0.0003	8.7	(23)

a Tp^–^ = trispyrazolylborate; L_1_ = ^t^BuPO(NH^*i*^Pr)_2_; L_2_ = 3,5-dinitrobenzoic acid; L_3_ = 2,4-dinitrobenzoic acid; H_2_quinha = quinaldic hydroxamic acid; CyPh_2_PO = cyclohexyl(diphenyl)phosphine oxide; BTC = 1,3,5-benzenetricarboxylate; fdh = 1,1,1-fluoro-5,5-dimethylhexa-2,4-dione; bpy = 2,2′-bipyridine; Cp^ttt^ = C_5_H_2_^*t*^Bu_3_-1,2,4; and COT″ = bis(trimethylsilyl)cyclooctatetraenyl dianion.

### Theoretical Studies

**Figure 7:**
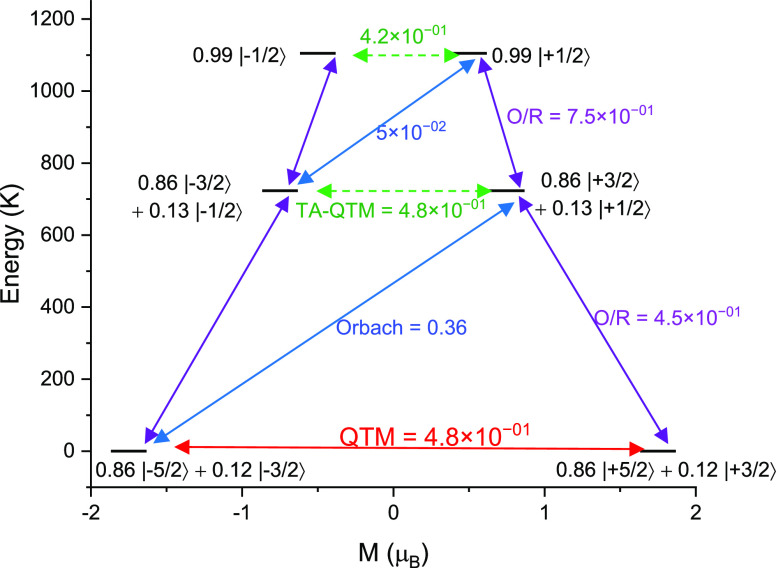
Energy-level distributions for **1**-Ce with the indicated probability of the relaxation path: QTM (red arrow) from where the actual relaxation in zero field occurs; Orbach (blue arrow); Orbach/Raman (purple arrow); and TA-QTM (TA = thermally assisted; green dashed arrow). The numbers above each arrow represent the corresponding transverse matrix elements for the transition magnetic moments.

Among the reported compounds, **1**-Ce, **3**-Nd, and **5**-Tb were analyzed using CASSCF, RASSI-SO, and SINGLE_ANISO calculations using the structures obtained from single-crystal X-ray diffraction as their inputs. For **1**-Ce, the three lowest lying Kramers doublets (KDs) span the range 0–1105 K, and the ground state *g* values are *g*_*xx*_ = 1.235, *g*_*yy*_ = 1.335, and *g*_*zz*_ = 3.295. The composition of the ground state is predominantly *m*_J_ = ±5/2, although there is mixing with the *m*_J_ = ±3/2 state (see Table 5). This suggests a stronger axial contribution to the crystal field than the equatorial contribution, which is in line with other Ce(III) SIMs previously studied by some of us.^32,51^ Furthermore, the orientation of the ground state *g*_*zz*_ axis coincides with the Ce–F bond (see Figure S19). However, the transverse anisotropy present does lead to some mixing of the ground state with excited states (see Table 5). This leads to ground-state QTM, consistent with the absence of slow relaxation of the magnetization in the absence of an applied dc field. The first excited state lies at 723 K (see Figure 7 and Table 5); hence, if the QTM is even partially quenched by using an applied dc field, **1**-Ce is likely to show slow relaxation of magnetization, as we observe experimentally.

**Table 5:** Energies (K), *m*_J_ Composition of the Lowest Doublets, and g-Tensors of the Individual Lanthanide Magnetic Centers Associated with Each State for **1**-Ce^a^

KD	energy (K)	composition |*m*_J_|	*g*_*xx*_	*g*_*yy*_	*g*_*zz*_	θ (deg)
1	0.0	0.86 |± 5/2⟩ + 0.12 |± 3/2⟩	1.235	1.335	3.295	
2	723.4	0.86 |± 3/2⟩ + 0.13 |± 1/2⟩	1.208	1.411	1.640	89.5
3	1104.7	0.99 |± 1/2⟩	2.695	2.242	0.641	1.6

a The angle between the ground-state *g*_*zz*_ and the respective excited-state *g*_zz_ axes is represented by θ.

The computed LoProp^73^ charge is −0.88 on the fluoride ion, whereas the combination of the nitrogen atoms in the equatorial plane yield total LoProp charges of −1.60 and −1.37 (considering two different sets of charges arising from the different nitrogen atoms, one for the nitrogen atoms in the aza-crown and another one for the nitrogen atoms in the pyridine groups) (see Table S8). Hence, there is a large contribution from the nitrogen atoms to the equatorial crystal field. It has been shown previously that, unlike Dy(III), Ce(III) ions are very sensitive to the equatorial ligand field,^51^ and this rationalizes our experimental observations. By considering the crystal field parameters (Table S9), it can be seen that although *B*_2_^0^ is larger than the *B*_2_^*q*^ non-axial components (where *q* ≠ 0), which disfavors QTM, the *B*_4_^0^ parameter is smaller than some of the *B*_4_^*q*^ non-axial components (where *q* ≠ 0), which favors QTM. Furthermore, other factors such as significant *g*_*xx*_/*g*_*yy*_ values and a mixed ground KD also promote QTM (Table 5).

**Figure 8:**
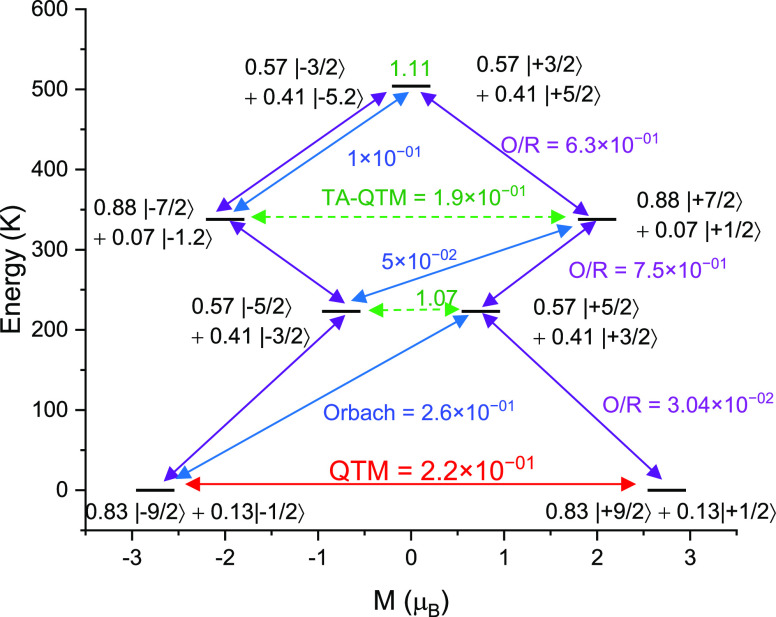
Energy-level distributions for **3**-Nd with the indicated probability of the relaxation path: QTM (red arrow) from where the actual relaxation in zero field occurs; Orbach (blue arrow); Orbach/Raman (purple arrow); and TA-QTM (TA = thermally assisted; green dashed arrow). The numbers above each arrow represent the corresponding transverse matrix elements for the transition magnetic moments.

For **3**-Nd, the computed ground-state g values are *g*_*xx*_ = 0.638, *g*_*yy*_ = 0.707, and *g*_*zz*_ = 5.513 (see Table 6). While the composition of the ground state is predominantly *m*_J_ = ±9/2, there is mixing with the *m*_J_ = ±1/2 state (see Table 6). The ground-state *g*_*zz*_ axis coincides with the Nd–F bond (Figure S19) as we saw for the Ce–F bond in **1**-Ce. However, the first excited KD in **3**-Nd lies at 223 K (see Table 6), which is far lower in energy than the first excited KD in **1**-Ce. This is reflected in a smaller *B*_2_^0^ parameter for **3**-Nd than for **1**-Ce (Table S9). Strong transverse anisotropy coupled with the mixing of the m_J_ states facilitates ground-state QTM in **3**-Nd, again suggesting that a zero-field SMM behavior should not be expected, as we observe experimentally. The relaxation mechanism calculated (see Figure 8) shows that if the ground-state QTM is quenched by a dc field, there is a possibility for slow relaxation of the magnetization, as we observe experimentally.^26,65,71^

**Table 6:** Energies (K), *m*_J_ Composition of Lowest Doublets, and g-Tensors of the Individual Lanthanide Magnetic Centers Associated with Each State for **3**-Nd^a^

KD	energy (K)	composition |*m*_J_|	*g*_*xx*_	*g*_*yy*_	*g*_*zz*_	θ (deg)
1	0.0	0.83 |± 9/2⟩ + 0.13 |± 1/2⟩	0.638	0.707	5.513	
2	223.3	0.57 |± 5/2⟩ + 0.41 |± 3/2⟩	3.521	2.911	1.175	1.2
3	337.8	0.88 |± 7/2⟩ + 0.07 |± 1/2⟩	0.439	0.722	4.252	0.04
4	503.9	0.57 |± 3/2⟩ + 0.41 |± 5/2⟩	3.812	2.880	0.268	0.8

a The angle between the ground-state *g*_*zz*_ and the respective excited-state *g*_zz_ axes is represented by θ.

The analogue **5**-Tb presents a different case to **1**-Ce and **3**-Nd, as Tb(III) is a non-Kramers ion. The *g* values of the ground state are calculated to be *g*_*xx*_ = 0.000, *g*_*yy*_ = 0.000, and *g*_*zz*_ = 17.818. The ground-state magnetic moment is aligned along the Tb–F bond, as in **1**-Ce and **3**-Nd (see Figure S19). However, a large tunnel splitting was calculated for **5**-Tb, and this is due to the position of the nitrogen donors from the ligand L, which provide a significant equatorial ligand field that enhances the tunnel splitting (see Table S10). Hence, applying a magnetic field is probably not enough to quench QTM, which agrees with the lack of any significant slow magnetic relaxation for **5**-Tb.

To understand further the magnetic properties of these complexes and the role of the fluoride ligand in promoting strong axiality, we have prepared a series of computational models **1**-Ce(a), **3**-Nd(a), and **5**-Tb(a) where the axial F^–^ ligand is removed, so that each model has just a {LnN_8_} coordination environment (see Figure S19).^74^ The model structures were optimized using DFT calculations (see Experimental Methods). For the **1**-Ce(a) and **3**-Nd(a) models, the calculations reveal smaller *g*_*zz*_ values and larger *g*_*xx*_ and *g*_*yy*_ values, with the excited KDs closer in energy (see Table 7). This highlights the importance of the F^–^ ligand in creating a strong axial crystal field for **1**-Ce and **3**-Nd and the observed slow relaxation of the magnetization.

**Table 7:** Energies (K) and Composition of *m*_J_ States of Lowest Doublets and g-Tensors of the Individual Lanthanide Magnetic Centers Associated with Each State for Model Complexes **1**-Ce(a), **1**-Ce(b), **3**-Nd(a), and **3**-Nd(b)^a^

	energy (K)	composition |*m*_J_ states|	*g*_*xx*_	*g*_*yy*_	*g*_*zz*_	θ (deg)
**1**-Ce(a)	0.00	0.99 |± 1/2⟩	2.873	2.238	0.810	
	396.2	0.82 |± 3/2⟩ + 0.17 |± 5/2⟩	1.696	1.457	1.042	88.9
	1121.0	0.82 |± 5/2⟩ + 0.17 |± 3/2⟩	1.327	1.422	3.037	0.6
**1**-Ce(b)	0.0	0.48 |± 5/2⟩ + 0.40 |± 3/2⟩	1.866	1.836	1.174	
	150.3	0.99 |± 1/2⟩	2.559	2.524	0.765	0.0
	511.2	0.55 |± 3/2⟩ + 0.44 |± 5/2⟩	1.906	1.891	0.509	0.0
**3**-Nd(a)	0.0	0.49 |± 5/2⟩ + 0.48 |± 3/2⟩	3.255	3.107	0.800	
	91.9	0.72 |± 1/2⟩ + 0.19 |± 9/2⟩ + 0.07 |± 7/2⟩	3.284	2.727	1.4103	0.3
	218.9	0.50 |± 3/2⟩ + 0.49 |± 5/2⟩	3.598	2.956	0.764	0.2
	329.8	0.89 |± 7/2⟩ + 0.09 |± 9/2⟩	0.997	1.375	4.046	1.0
**3**-Nd(b)	0.0	0.55 |± 5/2⟩ + 0.45 |± 3/2⟩	3.298	3.167	1.046	
	17.4	0.49 |± 9/2⟩ + 0.46 |± 1/2⟩ + 0.07 |± 7/2⟩	2.081	2.191	3.181	0.0
	193.8	0.85 |± 7/2⟩ 0.14 |± 9/2⟩	1.475	1.520	3.516	0.0
	243.7	0.55 |± 3/2⟩ 0.49 |± 5/2⟩	3.349	3.346	0.456	0.0

a The angle between ground-state *g*_*zz*_ and the respective excited-state *g*_*zz*_ axes is represented by θ.

**Figure 9:**
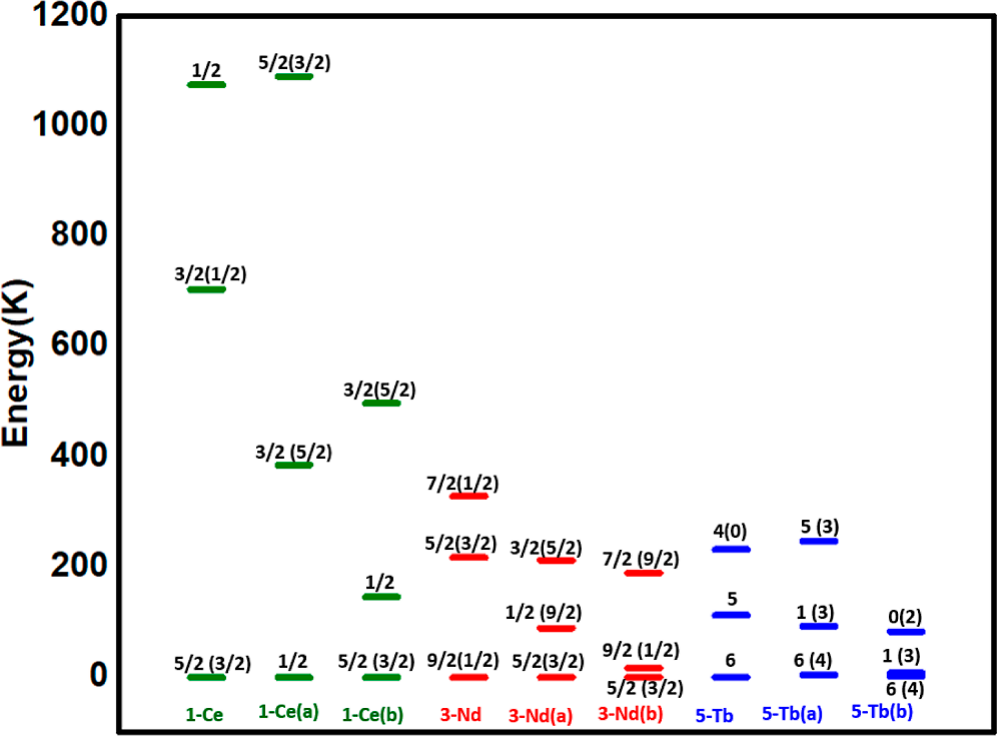
Comparative energies (in K) of the first three *m*_J_ states in **1**-Ce, **1**-Ce(a), and **1**-Ce(b); **3**-Nd, **3**-Nd(a), and **3**-Nd(b); and **5**-Tb, **5**-Tb(a), and **5**-Tb(b). Models (a) have the axial F^–^ ligand removed and models (b) have the axial F^–^ replaced by I^–^. The major composition of the m_J_ states is shown, with the largest minor contribution given in brackets.

The ground m_J_ state is found to be ±1/2 for the model **1**-Ce(a) and a mixture of ±5/2 and ±3/2 for the model **3**-Nd(a) (see Figure 9). Hence, upon removal of the F^–^ ligand, the nature of the ground state changes, with the [N_8_] ligand stabilizing instead m_J_ states with stronger prolate 4f charge density for **1**-Ce(a) and **3**-Nd(a). For the model **5**-Tb(a), the tunnel splitting is increased further after the removal of the F^–^ ligand (see Table S10). Furthermore, in **5**-Tb(a), the direction of the ground-state *g*_*zz*_ axis passes through the direction that bisects the plane formed by the pyridine and aza-crown nitrogen atoms (see Figure S19). This shows that the F^–^ ligand is essential to align the *g*_*zz*_ axis along the pseudo-*C*_4_ axis for **5**-Tb. Furthermore, the ground-state to first-excited-state gap reduces upon removal of the F^–^ ligand, and the energy states are extremely mixed, although the ground m_J_ state is still predominantly ±6, which has an oblate charge density. We note that eight nitrogen donor atoms present in a pseudo-*D*_4*d*_ environment are known to stabilize the *m*_J_ = ± 6 state, for example, in [Tb(pc)_2_]^−^, which has a sandwich-like ligand arrangement.^11^

We also have modeled another set of molecules, **1**-Ce(b), **3**-Nd(b), and **5**-Tb(b), where the axial F^–^ ligand is replaced by an I^–^ ligand. The model structures were optimized using DFT calculations (see Experimental Methods). The optimized Ln-I distance is 3.3 Å, compared to the Ln-F distance of 2.2 Å (see Table 1). The orientation of the *g*_*zz*_ axis is along the pseudo-*C*_4_ axis in **1**-Ce(b) and **3**-Nd(b) as in **1**-Ce and **3**-Nd, that is, along the Ln–I bond (Figure S19). However, for these models, there is a reduction in the axial crystal field: the energy gap between the first KD and the second KD, which was 723 K in **1-**Ce is lowered to 150 K for **1-**Ce(b), while the 223 K gap in **3-**Nd decreases to 17 K in **3-**Nd(b) (see Table 7 and Figure 9). Again, this highlights the importance of the F^–^ ligand in creating a strong axial crystal field for **1**-Ce and **3**-Nd. In model **5**-Tb(b), there is also a significant tunnel splitting (see Table S10); however, the first exited state is very low in energy (7.1/8.6 K). In this model, the *g*_*zz*_ axis lies in the plane between the pyridine and aza-crown nitrogens, as it did in model **5**-Tb(a), reflecting the fact that the I^–^ ligand does not provide a sufficient axial crystal field to offset the eight nitrogen atoms in a *D*_4*d*_ environment. Here, we can draw parallels to earlier work on Na[Tb^III^(DOTA)(H_2_O)]·4H_2_O (H_4_DOTA is 1,4,7,10-tetraazacyclododecane-1,4,7,10-tetraacetic acid), which has a weak axial H_2_O ligand, where the easy axis is found perpendicular to the Ln–H_2_O bond.^75^

## Conclusions

In summary, we report the structural and magnetic study of a family of lanthanide compounds featuring an axial Ln–F bond, including eight new analogues (**1**-Ce, **2**-Pr, **3**-Nd, **5**-Tb, **6**-Ho, **7**-Er, **8**-Tm, and **9**-Yb). From these, the **1**-Ce and **3**-Nd analogues show slow relaxation of the magnetization under an applied dc field of 1200 and 800 Oe, respectively, which is modeled using a Raman process. The strong axial magnetic anisotropy generated by the fluoride ligand helps promote the SMM behavior in the oblate lanthanides Ce(III) and Nd(III), both Kramers ions, and the relaxation pathways have been elucidated by performing *ab initio* calculations. The Tb(III) complex does not show any significant slow relaxation of the magnetization, even when diluted with Y. We have shown that this can be attributed to a large tunnel splitting in the ground state and the non-Kramers nature of the ion. Furthermore, the analysis of **1**-Ce(a), **3-**Nd(a), and **5**-Tb(a) model complexes, where the axial fluoride ligand is removed to study the effect of the [N_8_] coordination cage, and **1**-Ce(b), **3**-Nd(b), and **5**-Tb(b), where the F^–^ is replaced by a I^–^, show that the crystal field splitting is dramatically reduced. This highlights the importance of the F^–^ ligand in creating a strong axial crystal field for **1**-Ce and **3**-Nd and for promoting the SMM behavior.

## Supporting Information Available

Ligand ^1^H NMR; crystallographic tables; crystal packing diagrams; PXRD patterns; further magnetic data; and further computational studies (PDF)

## References

[ref1] Lis T. Preparation, structure, and magnetic properties of a dodecanuclear mixed-valence manganese carboxylate. Acta Crystallogr., Sect. B: Struct. Crystallogr. Cryst. Chem. 1980, 36, 2042–2046. 10.1107/s0567740880007893.

[ref2] Sessoli R.; Gatteschi D.; Caneschi A.; Novak M. A. Magnetic bistability in a metal-ion cluster. Nature 1993, 365, 141–143. 10.1038/365141a0.

[ref3] Sessoli R.; Tsai H. L.; Schake A. R.; Wang S.; Vincent J. B.; Folting K.; Gatteschi D.; Christou G.; Hendrickson D. N. High-spin molecules: [Mn12O12(O2CR)16(H2O)4]. J. Am. Chem. Soc. 1993, 115, 1804–1816. 10.1021/ja00058a027.

[ref4] Caneschi A.; Gatteschi D.; Sessoli R.; Barra A. L.; Brunel L. C.; Guillot M. Alternating current susceptibility, high field magnetization, and millimeter band EPR evidence for a ground S = 10 state in [Mn12O12(Ch3COO)16(H2O)4].2CH3COOH.4H2O. J. Am. Chem. Soc. 1991, 113, 5873–5874. 10.1021/ja00015a057.

[ref5] Milios C. J.; Raptopoulou C. P.; Terzis A.; Lloret F.; Vicente R.; Perlepes S. P.; Escuer A. Hexanuclear Manganese(III) Single-Molecule Magnets. Angew. Chem., Int. Ed. 2004, 43, 210–212. 10.1002/anie.200351079. 14695611

[ref6] Milios C. J.; Vinslava A.; Whittaker A. G.; Parsons S.; Wernsdorfer W.; Christou G.; Perlepes S. P.; Brechin E. K. Microwave-Assisted Synthesis of a Hexanuclear MnIII Single-Molecule Magnet. Inorg. Chem. 2006, 45, 5272–5274. 10.1021/ic0606678. 16813386

[ref7] Gatteschi D.; Sessoli R.; Cornia A. Single-molecule magnets based on iron(III) oxo clusters. Chem. Commun. 2000, 725–732. 10.1039/a908254i.

[ref8] Barra A. L.; Caneschi A.; Cornia A.; Fabrizi de Biani F.; Gatteschi D.; Sangregorio C.; Sessoli R.; Sorace L. Single-Molecule Magnet Behavior of a Tetranuclear Iron(III) Complex. The Origin of Slow Magnetic Relaxation in Iron(III) Clusters. J. Am. Chem. Soc. 1999, 121, 5302–5310. 10.1021/ja9818755.

[ref9] Cadiou C.; Murrie M.; Paulsen C.; Villar V.; Wernsdorfer W.; Winpenny R. E. P. Studies of a nickel-based single molecule magnet: resonant quantum tunnelling in an S = 12 molecule. Chem. Commun. 2001, 2666–2667. 10.1039/b108894g.

[ref10] Murrie M.; Teat S. J.; Stœckli-Evans H.; Güdel H. U. Synthesis and Characterization of a Cobalt(II) Single-Molecule Magnet. Angew. Chem., Int. Ed. 2003, 42, 4653–4656. 10.1002/anie.200351753. 14533155

[ref11] Ishikawa N.; Sugita M.; Ishikawa T.; Koshihara S.-Y.; Kaizu Y. Lanthanide Double-Decker Complexes Functioning as Magnets at the Single-Molecular Level. J. Am. Chem. Soc. 2003, 125, 8694–8695. 10.1021/ja029629n. 12862442

[ref12] Guo F.-S.; Day B. M.; Chen Y.-C.; Tong M.-L.; Mansikkamäki A.; Layfield R. A. Magnetic hysteresis up to 80 kelvin in a dysprosium metallocene single-molecule magnet. Science 2018, 362, 1400–1403. 10.1126/science.aav0652. 30337456

[ref13] Gonidec M.; Amabilino D. B.; Veciana J. Novel double-decker phthalocyaninato terbium(III) single molecule magnets with stabilised redox states. Dalton Trans. 2012, 41, 13632–13639. 10.1039/c2dt31171b. 22878265

[ref14] Shintoyo S.; Murakami K.; Fujinami T.; Matsumoto N.; Mochida N.; Ishida T.; Sunatsuki Y.; Watanabe M.; Tsuchimoto M.; Mrozinski J.; Coletti C.; Re N. Crystal field splitting of the ground state of terbium(III) and dysprosium(III) complexes with a triimidazolyl tripod ligand and an acetate determined by magnetic analysis and luminescence. Inorg. Chem. 2014, 53, 10359–10369. 10.1021/ic501453h. 25203929

[ref15] Chen Y.-C.; Liu J.-L.; Wernsdorfer W.; Liu D.; Chibotaru L. F.; Chen X.-M.; Tong M.-L. Hyperfine-Interaction-Driven Suppression of Quantum Tunneling at Zero Field in a Holmium(III) Single-Ion Magnet. Angew. Chem., Int. Ed. 2017, 129, 5078–5082. 10.1002/ange.201701480. 28295930

[ref16] Feltham H. L. C.; Brooker S. Review of purely 4f and mixed-metal nd-4f single-molecule magnets containing only one lanthanide ion. Coord. Chem. Rev. 2014, 276, 1–33. 10.1016/j.ccr.2014.05.011.

[ref17] Gould C. A.; McClain K. R.; Yu J. M.; Groshens T. J.; Furche F.; Harvey B. G.; Long J. R. Synthesis and Magnetism of Neutral, Linear Metallocene Complexes of Terbium(II) and Dysprosium(II). J. Am. Chem. Soc. 2019, 141, 12967–12973. 10.1021/jacs.9b05816. 31375028

[ref18] Rinehart J. D.; Long J. R. Exploiting single-ion anisotropy in the design of f-element single-molecule magnets. Chem. Sci. 2011, 2, 2078–2085. 10.1039/c1sc00513h.

[ref19] Ungur L.; Chibotaru L. F. Strategies toward High-Temperature Lanthanide-Based Single-Molecule Magnets. Inorg. Chem. 2016, 55, 10043–10056. 10.1021/acs.inorgchem.6b01353. 27508399

[ref20] Briganti M.; Santanni F.; Tesi L.; Totti F.; Sessoli R.; Lunghi A. A Complete Ab Initio View of Orbach and Raman Spin-Lattice Relaxation in a Dysprosium Coordination Compound. J. Am. Chem. Soc. 2021, 143, 13633–13645. 10.1021/jacs.1c05068. 34465096 PMC8414553

[ref21] Giansiracusa M. J.; Kostopoulos A. K.; Collison D.; Winpenny R. E. P.; Chilton N. F. Correlating blocking temperatures with relaxation mechanisms in monometallic single-molecule magnets with high energy barriers (Ueff > 600 K). Chem. Commun. 2019, 55, 7025–7028. 10.1039/c9cc02421b. 31066737

[ref22] Rousset E.; Piccardo M.; Boulon M. E.; Gable R. W.; Soncini A.; Sorace L.; Boskovic C. Slow Magnetic Relaxation in Lanthanoid Crown Ether Complexes: Interplay of Raman and Anomalous Phonon Bottleneck Processes. Chem.—Eur. J. 2018, 24, 14768–14785. 10.1002/chem.201802779. 29992641

[ref23] Liu J.; Reta D.; Cleghorn J. A.; Yeoh Y. X.; Ortu F.; Goodwin C. A. P.; Chilton N. F.; Mills D. P. Light Lanthanide Metallocenium Cations Exhibiting Weak Equatorial Anion Interactions. Chem.—Eur. J. 2019, 25, 7749–7758. 10.1002/chem.201901167. 30994214 PMC6637382

[ref24] Gu L.; Wu R. Origins of Slow Magnetic Relaxation in Single-Molecule Magnets. Phys. Rev. Lett. 2020, 125, 117203 10.1103/physrevlett.125.117203. 32975970

[ref25] Li Q.-W.; Wan R.-C.; Chen Y.-C.; Liu J.-L.; Wang L.-F.; Jia J.-H.; Chilton N. F.; Tong M.-L. Unprecedented hexagonal bipyramidal single-ion magnets based on metallacrowns. Chem. Commun. 2016, 52, 13365–13368. 10.1039/c6cc06924j. 27785484

[ref26] Chen Y.-C.; Huang X.-S.; Liu J.-L.; Tong M.-L. Magnetic Dynamics of a Neodymium(III) Single-Ion Magnet. Inorg. Chem. 2018, 57, 11782–11787. 10.1021/acs.inorgchem.8b01957. 30160953

[ref27] Lunghi A.; Sanvito S. The Limit of Spin Lifetime in Solid-State Electronic Spins. J. Phys. Chem. Lett. 2020, 11, 6273–6278. 10.1021/acs.jpclett.0c01681. 32667205

[ref28] Escalera-Moreno L.; Baldoví J. J.; Gaita-Ariño A.; Coronado E. Spin states, vibrations and spin relaxation in molecular nanomagnets and spin qubits: a critical perspective. Chem. Sci. 2018, 9, 3265–3275. 10.1039/c7sc05464e. 29780458 PMC5935026

[ref29] Wada H.; Ooka S.; Yamamura T.; Kajiwara T. Light Lanthanide Complexes with Crown Ether and Its Aza Derivative Which Show Slow Magnetic Relaxation Behaviors. Inorg. Chem. 2017, 56, 147–155. 10.1021/acs.inorgchem.6b01764. 27936628

[ref30] Gupta S. K.; Rajeshkumar T.; Rajaraman G.; Murugavel R. An air-stable Dy(III) single-ion magnet with high anisotropy barrier and blocking temperature. Chem. Sci. 2016, 7, 5181–5191. 10.1039/c6sc00279j. 30155168 PMC6020529

[ref31] Gupta S. K.; Rajeshkumar T.; Rajaraman G.; Murugavel R. An unprecedented zero field neodymium(III) single-ion magnet based on a phosphonic diamide. Chem. Commun. 2016, 52, 7168–7171. 10.1039/c6cc03066a. 27173026

[ref32] Gupta S. K.; Shanmugan S.; Rajeshkumar T.; Borah A.; Damjanović M.; Schulze M.; Wernsdorfer W.; Rajaraman G.; Murugavel R. A single-ion single-electron cerrous magnet. Dalton Trans. 2019, 48, 15928–15935. 10.1039/c9dt03052b. 31513208

[ref33] Canaj A. B.; Singh M. K.; Regincós Marti E.; Damjanović M.; Wilson C.; Céspedes O.; Wernsdorfer W.; Rajaraman G.; Murrie M. Boosting axiality in stable high-coordinate Dy(III) single-molecule magnets. Chem. Commun. 2019, 55, 5950–5953. 10.1039/c9cc00965e. 31049540

[ref34] Norel L.; Darago L. E.; Le Guennic B.; Chakarawet K.; Gonzalez M. I.; Olshansky J. H.; Rigaut S.; Long J. R. A Terminal Fluoride Ligand Generates Axial Magnetic Anisotropy in Dysprosium Complexes. Angew. Chem., Int. Ed. 2018, 57, 1933–1938. 10.1002/anie.201712139. 29285845

[ref35] Tsukube H.; Mizutani Y.; Shinoda S.; Okazaki T.; Tadokoro M.; Hori K. Side Arm Effects on Cyclen–Alkali Metal Cation Complexation: Highly Selective and Three-Dimensional Encapsulation of Na+ Ion. Inorg. Chem. 1999, 38, 3506–3512. 10.1021/ic980590q. 11671097

[ref36] Bu X.-H.; Cao X.-C.; Zhang W.-Q.; Zhang R.-H.; Clifford T. A new tetraazamacrocycle functionalized with pendant pyridyl groups: synthesis and crystal structure of a copper(II) complex of 1,4,7,10-tetrakis(2-pyridylmethyl)-1,4,7,10-tetraazacyclododecane (L), [CuL]·(ClO_4_)_2_. Transition Met. Chem. 1997, 22, 513–515. 10.1023/a:1018527716715.

[ref37] Bu X.-H.; Cao X.-C.; Chen W.; Zhang R.-H.; Thomas C. A new tetraazamacrocycle functionalized with four pendant pyridyl groups: synthesis and crystal structure of the nickel(II) complex of 1,4,7,10-tetrakis(2-pyridylmethyl)-1,4,7,10-tetraazacyclododecane(L), [NiL]2+. Polyhedron 1998, 17, 289–293. 10.1016/s0277-5387(97)00305-7.

[ref38] Bu X.-H.; Chen W.; Mu L.-J.; Zhang Z.-H.; Zhang R.-H.; Clifford T. Syntheses, crystal structures and properties of new manganese(II) complexes with macrocyclic polyamine ligands bearing pyridyl donor pendants. Polyhedron 2000, 19, 2095–2100. 10.1016/s0277-5387(00)00510-6.

[ref39] Bu X.-H.; Lu S.-L.; Zhang R.-H.; Liu H.; Zhu H.-P.; Liu Q.-T. Synthesis, characterization and crystal structures of the cobalt(II) and iron(II) complexes with an octadentate ligand, 1,4,7,10-tetrakis(2-pyridylmethyl)-1,4,7,10-tetraazacyclododecane (L), [ML]2+. Polyhedron 2000, 19, 431–435. 10.1016/s0277-5387(99)00381-2.

[ref40] Morfin J.-F.; Tripier R.; Baccon M. L.; Handel H. Bismuth(III) complexes with tetra-pyridylmethyl-cyclen. Inorg. Chem. Acta 2009, 362, 1781–1786. 10.1016/j.ica.2008.08.013.

[ref41] Ito H.; Tsukube H.; Shinoda S. Chirality Transfer in Propeller-Shaped Cyclen–Calcium(II) Complexes: Metal-Coordinating and Ion-Pairing Anion Procedures. Chem.—Eur. J. 2013, 19, 3330–3339. 10.1002/chem.201204323. 23404763

[ref42] Wada A.; Watanabe M.; Yamanoi Y.; Nankawa T.; Namiki K.; Yamasaki M.; Murata M.; Nishihara H. Control of Coordination and Luminescence Properties of Lanthanide Complexes Using Octadentate Oligopyridine-Amine Ligands. Bull. Chem. Soc. Jpn. 2007, 80, 335–345. 10.1246/bcsj.80.335.

[ref43] Misaki H.; Miyake H.; Shinoda S.; Tsukube H. Asymmetric Twisting and Chirality Probing Properties of Quadruple-Stranded Helicates: Coordination Versatility and Chirality Response of Na+, Ca2+, and La3+ Complexes with Octadentate Cyclen Ligand. Inorg. Chem. 2009, 48, 11921–11928. 10.1021/ic901496s. 19919024

[ref44] Natrajan L. S.; Khoabane N. M.; Dadds B. L.; Muryn C. A.; Pritchard R. G.; Heath S. L.; Kenwright A. M.; Kuprov I.; Faulkner S. Probing the structure, conformation, and stereochemical exchange in a family of lanthanide complexes derived from tetrapyridyl-appended cyclen. Inorg. Chem. 2010, 49, 7700–7709. 10.1021/ic100447m. 20799736

[ref45] Wilson J. J.; Birnbaum E. R.; Batista E. R.; Martin R. L.; John K. D. Synthesis and Characterization of Nitrogen-Rich Macrocyclic Ligands and an Investigation of Their Coordination Chemistry with Lanthanum(III). Inorg. Chem. 2015, 54, 97–109. 10.1021/ic501843c. 25526533

[ref46] Blackburn O. A.; Kenwright A. M.; Jupp A. R.; Goicoechea J. M.; Beer P. D.; Faulkner S. Fluoride Binding and Crystal-Field Analysis of Lanthanide Complexes of Tetrapicolyl-Appended Cyclen. Chem.—Eur. J. 2016, 22, 8929–8936. 10.1002/chem.201601170. 27167830

[ref47] Wada A.; Watanabe M.; Yamanoi Y.; Nishihara H. Modification of the luminescence spectra of chloro(tetrapyridylcyclotetramine)europium complexes by fine tuning of the Eu-Cl distance with outer-sphere counterions in the solid state, in a polymer matrix and in solution. Chem. Commun. 2008, 1671–1673. 10.1039/b716987f. 18368160

[ref48] Aquilante F.; Autschbach J.; Carlson R. K.; Chibotaru L. F.; Delcey M. G.; De Vico L.; Fdez. Galván I.; Ferré N.; Frutos L. M.; Gagliardi L.; Garavelli M.; Giussani A.; Hoyer C. E.; Li Manni G.; Lischka H.; Ma D.; Malmqvist P. Å.; Müller T.; Nenov A.; Olivucci M.; Pedersen T. B.; Peng D.; Plasser F.; Pritchard B.; Reiher M.; Rivalta I.; Schapiro I.; Segarra-Martí J.; Stenrup M.; Truhlar D. G.; Ungur L.; Valentini A.; Vancoillie S.; Veryazov V.; Vysotskiy V. P.; Weingart O.; Zapata F.; Lindh R. Molcas 8: New capabilities for multiconfigurational quantum chemical calculations across the periodic table. J. Comput. Chem. 2016, 37, 506–541. 10.1002/jcc.24221. 26561362

[ref49] Chibotaru L. F.; Ungur L. Ab initio calculation of anisotropic magnetic properties of complexes. I. Unique definition of pseudospin Hamiltonians and their derivation. J. Chem. Phys. 2012, 137, 064112 10.1063/1.4739763. 22897260

[ref50] Granovsky A. A. Extended multi-configuration quasi-degenerate perturbation theory: The new approach to multi-state multi-reference perturbation theory. J. Chem. Phys. 2011, 134, 214113 10.1063/1.3596699. 21663350

[ref51] Singh S. K.; Gupta T.; Ungur L.; Rajaraman G. Magnetic Relaxation in Single-Electron Single-Ion Cerium(III) Magnets: Insights from Ab Initio Calculations. Chem.—Eur. J. 2015, 21, 13812–13819. 10.1002/chem.201501330. 26262751

[ref52] Malmqvist P. Å.; Roos B. O.; Schimmelpfennig B. The restricted active space (RAS) state interaction approach with spin-orbit coupling. Chem. Phys. Lett. 2002, 357, 230–240. 10.1016/s0009-2614(02)00498-0.

[ref53] Stephens P. J.; Devlin F. J.; Chabalowski C. F.; Frisch M. J. Ab Initio Calculation of Vibrational Absorption and Circular Dichroism Spectra Using Density Functional Force Fields. J. Phys. Chem. 1994, 98, 11623–11627. 10.1021/j100096a001.

[ref54] Lee C.; Yang W.; Parr R. G. Development of the Colle-Salvetti correlation-energy formula into a functional of the electron density. Phys. Rev. B: Condens. Matter Mater. Phys. 1988, 37, 785–789. 10.1103/physrevb.37.785. 9944570

[ref55] Dolg M.; Wedig U.; Stoll H.; Preuss H. Energy-adjustedabinitiopseudopotentials for the first row transition elements. J. Chem. Phys. 1987, 86, 866–872. 10.1063/1.452288.

[ref56] Andrae D.; Häußermann U.; Dolg M.; Stoll H.; Preuß H. Energy-adjustedab initio pseudopotentials for the second and third row transition elements. Theor. Chim. Acta 1990, 77, 123–141. 10.1007/bf01114537.

[ref57] Frisch M. J.; Trucks G. W.; Schlegel H. B.; Scuseria G. E.; Robb M. A.; Cheeseman J. R.; Scalmani G.; Barone V.; Mennucci B.; Petersson G. A.; Nakatsuji H.; Caricato M.; Li X.; Hratchian H. P.; Izmaylov A. F.; Bloino J.; Zheng G.; Sonnenberg J. L.; Hada M.; Ehara M.; Toyota K.; Fukuda R.; Hasegawa J.; Ishida M.; Nakajima T.; Honda Y.; Kitao O.; Nakai H.; Vreven T.; Montgomery J. A. Jr; Peralta J. E.; Ogliaro F.; Bearpark M.; Heyd J. J.; Brothers E.; Kudin K. N.; Staroverov V. N.; Kobayashi R.; Normand J.; Raghavachari K.; Rendell A.; Burant J. C.; Iyengar S. S.; Tomasi J.; Cossi M.; Rega N.; Millam J. M.; Klene M.; Knox J. E.; Cross J. B.; Bakken V.; Adamo C.; Jaramillo J.; Gomperts R.; Stratmann R. E.; Yazyev O.; Austin A. J.; Cammi R.; Pomelli C.; Ochterski J. W.; Martin R. L.; Morokuma K.; Zakrzewski V. G.; Voth G. A.; Salvador P.; Dannenberg J. J.; Dapprich S.; Daniels A. D.; Farkas O.; Foresman J. B.; Ortiz J. V.; Cioslowski J.; Fox D. J. Gaussian 09, Revision A.02.; Gaussian, Inc.: Wallingford CT, 2009.

[ref58] Sievers J. Asphericity of 4f-shells in their Hund’s rule ground states. Z. Phys. B: Condens. Matter 1982, 45, 289–296. 10.1007/bf01321865.

[ref59] Pinsky M.; Avnir D. Continuous Symmetry Measures. 5. The Classical Polyhedra. Inorg. Chem. 1998, 37, 5575–5582. 10.1021/ic9804925. 11670704

[ref60] Ruiz-Martínez A.; Casanova D.; Alvarez S. Polyhedral structures with an odd number of vertices: nine-atom clusters and supramolecular architectures. Dalton Trans. 2008, 2583–2591. 10.1039/b718821h. 18443701

[ref61] Ruiz-Martínez A.; Casanova D.; Alvarez S. Polyhedral Structures with an Odd Number of Vertices: Nine-Coordinate Metal Compounds. Chem.—Eur. J. 2008, 14, 1291–1303. 10.1002/chem.200701137. 18000919

[ref62] Benelli C.; Gatteschi D. Introduction to Molecular Magnetism; Wiley-VCH, 2015.

[ref63] Blackburn O. A.; Edkins R. M.; Faulkner S.; Kenwright A. M.; Parker D.; Rogers N. J.; Shuvaev S. Electromagnetic susceptibility anisotropy and its importance for paramagnetic NMR and optical spectroscopy in lanthanide coordination chemistry. Dalton Trans. 2016, 45, 6782–6800. 10.1039/c6dt00227g. 26898996

[ref64] Parker D.; Suturina E. A.; Kuprov I.; Chilton N. F. How the Ligand Field in Lanthanide Coordination Complexes Determines Magnetic Susceptibility Anisotropy, Paramagnetic NMR Shift, and Relaxation Behavior. Acc. Chem. Res. 2020, 53, 1520–1534. 10.1021/acs.accounts.0c00275. 32667187 PMC7467575

[ref65] Jassal A. K.; Aliaga-Alcalde N.; Corbella M.; Aravena D.; Ruiz E.; Hundal G. Neodymium 1D systems: targeting new sources for field-induced slow magnetization relaxation. Dalton Trans. 2015, 44, 15774–15778. 10.1039/c5dt02533h. 26299199

[ref66] Sun L.; Zhang S.; Qiao C.; Chen S.; Yin B.; Wang W.; Wei Q.; Xie G.; Gao S. Fine-Tuning of the Coordination Environment To Regulate the Magnetic Behavior in Solvent/Anion-Dependent DyIII Compounds: Synthesis, Structure, Magnetism, and Ab Initio Calculations. Inorg. Chem. 2016, 55, 10587–10596. 10.1021/acs.inorgchem.6b01803. 27681198

[ref67] Reta D.; Chilton N. F. Uncertainty estimates for magnetic relaxation times and magnetic relaxation parameters. Phys. Chem. Chem. Phys. 2019, 21, 23567–23575. 10.1039/c9cp04301b. 31620733

[ref68] Baldoví J. J.; Clemente-Juan J. M.; Coronado E.; Duan Y.; Gaita-Ariño A.; Giménez-Saiz C. Construction of a General Library for the Rational Design of Nanomagnets and Spin Qubits Based on Mononuclear f-Block Complexes. The Polyoxometalate Case. Inorg. Chem. 2014, 53, 9976–9980. 10.1021/ic501867d. 25156530

[ref69] Le Roy J. J.; Korobkov I.; Kim J. E.; Schelter E. J.; Murugesu M. Structural and magnetic conformation of a cerocene [Ce(COT′)2]– exhibiting a uniconfigurational f1 ground state and slow-magnetic relaxation. Dalton Trans. 2014, 43, 2737–2740. 10.1039/c3dt53280a. 24366363

[ref70] Xu M.-X.; Meng Y.-S.; Xiong J.; Wang B.-W.; Jiang S.-D.; Gao S. Magnetic anisotropy investigation on light lanthanide complexes. Dalton Trans. 2018, 47, 1966–1971. 10.1039/c7dt04351a. 29344590

[ref71] Rinehart J. D.; Long J. R. Slow magnetic relaxation in homoleptic trispyrazolylborate complexes of neodymium(III) and uranium(III). Dalton Trans. 2012, 41, 13572–13574. 10.1039/c2dt31352a. 22878433

[ref72] Zhang H.-L.; Wu X.-Y.; Liao J.-Z.; Kuang X.-F.; Yang W.; Lu C.-Z. A novel trigonal propeller-shaped hybrid tri-neodymium-polyoxometalate exhibiting single-molecule magnet behavior. Dalton Trans. 2018, 47, 1796–1800. 10.1039/c7dt04908k. 29354842

[ref73] Gagliardi L.; Lindh R.; Karlström G. Local properties of quantum chemical systems: the LoProp approach. J. Chem. Phys. 2004, 121, 4494–4500. 10.1063/1.1778131. 15332879

[ref74] Gupta T.; Rajaraman G. Magnetic Anisotropy, Magneto-Structural Correlations and Mechanism of Magnetic Relaxation in {Dy III N 8 } Complexes: A Theoretical Perspective. Eur. J. Inorg. Chem. 2018, 3402–3412. 10.1002/ejic.201800350.

[ref75] Boulon M.-E.; Cucinotta G.; Luzon J.; Degl’Innocenti C.; Perfetti M.; Bernot K.; Calvez G.; Caneschi A.; Sessoli R. Magnetic Anisotropy and Spin-Parity Effect Along the Series of Lanthanide Complexes with DOTA. Angew. Chem., Int. Ed. 2013, 52, 350–354. 10.1002/anie.201205938. 23208792

