# Supplementary material for: Importance of an Axial LnIII–F Bond across the Lanthanide Series and Single-Molecule Magnet Behavior in the Ce and Nd Analogues
Source: Inorg Chem. 2022 Jun 21;61(26):9906–17. doi: 10.1021/acs.inorgchem.2c00556 (PMC9275778; doi:10.1021/acs.inorgchem.2c00556)
Supplement: Supplementary file 1 — ic2c00556_si_001.pdf [file ic2c00556_si_001.pdf]

SUPPORTING INFORMATION

The importance of an axial Ln^{III}-F bond across the lanthanide series and single-molecule magnet behavior in the Ce and Nd analogues

Emma Regincós Martí,[a] Angelos B. Canaj,[a, †,] Tanu Sharma,[b] Anna Celmina,[a] Claire Wilson,[a] Gopalan Rajaraman,[b,*] and Mark Murrie[a,*]*

[a] School of Chemistry, University of Glasgow, Glasgow, University Avenue G12 8QQ, United Kingdom.

[b] Department of Chemistry, Indian Institute of Technology Bombay, Powai, Mumbai, Maharashtra, 400076, India.

[†] Current address: Department of Chemistry, University of Liverpool, Liverpool, Crown Street, L69 7ZD, United Kingdom.

* mark.murrie@glasgow.ac.uk; rajaraman@chem.iitb.ac.in; Angelos.Tsanai@liverpool.ac.uk

Table of Contents

1. Synthesis and characterization S3

2. Magnetic characterization S16

3. *Ab initio* calculations S26

1. Synthesis and characterization

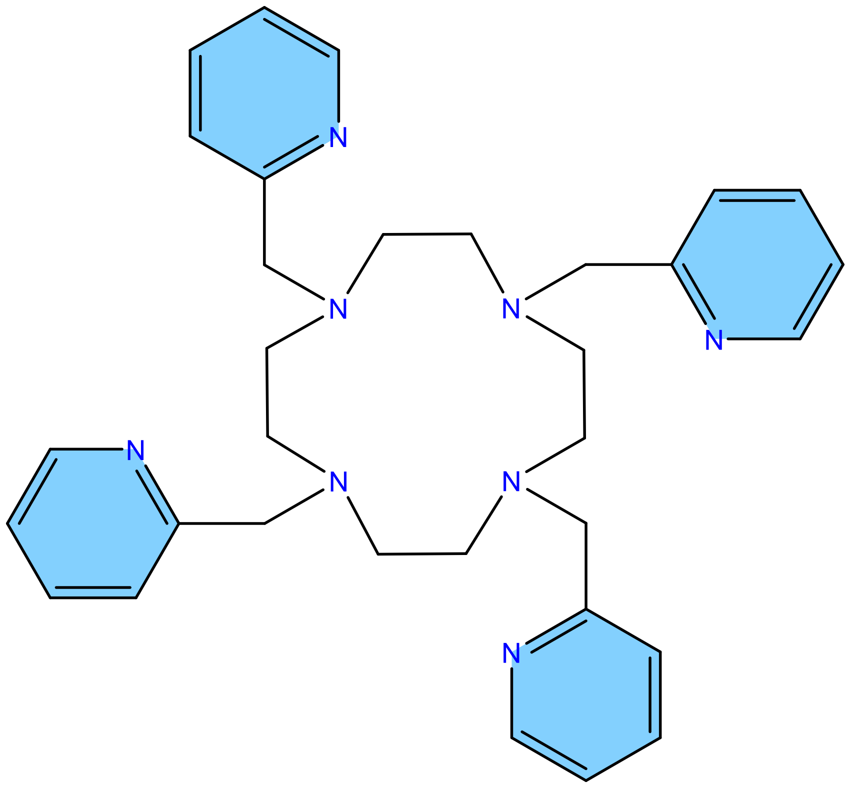

Figure S1. Schematic representation of the ligand 1,4,7,10-tetrakis(2-pyridylmethyl)-1,4,7,10-tetraaza-cyclododecane, L.

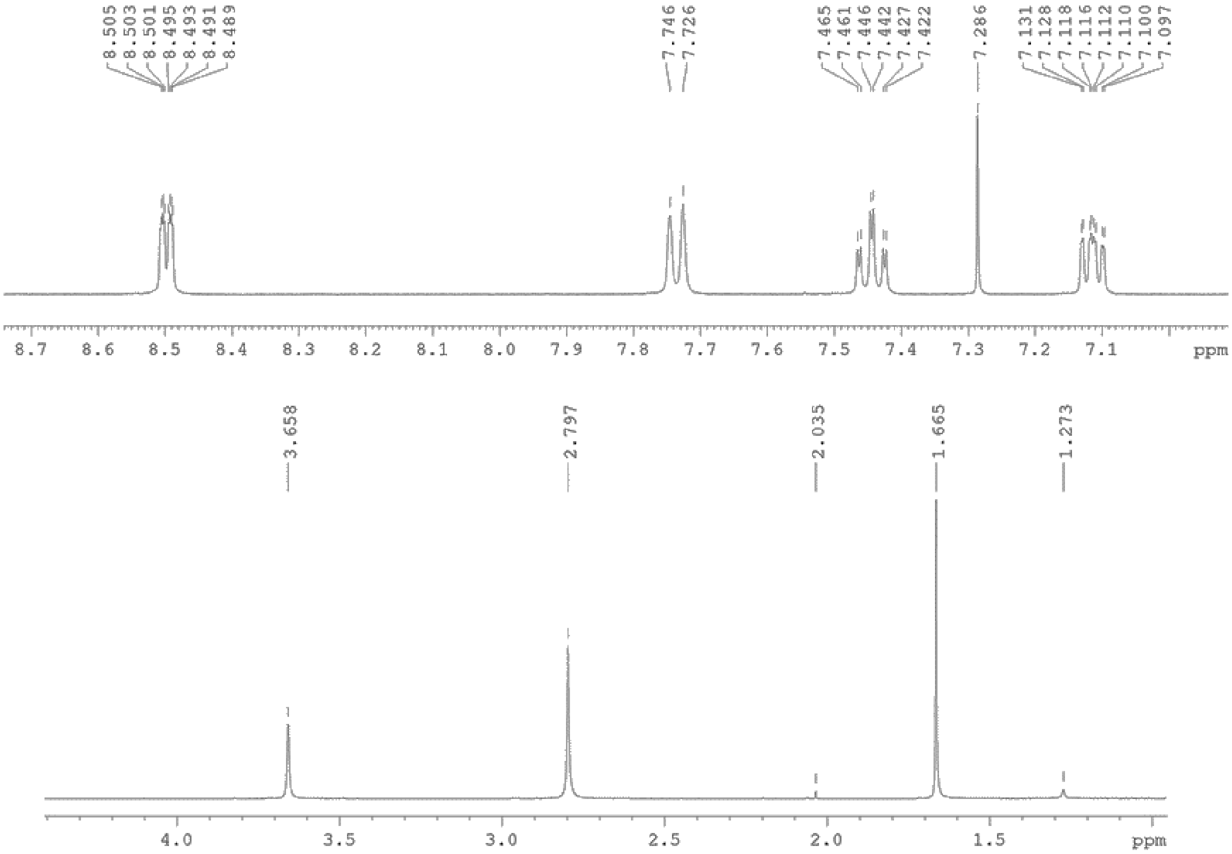

Figure S2. ^{1}H NMR of L in $CDCl_3$ at 298 K.

Table S1. Crystallographic information for **1**-Ce, **2**-Pr and **3**-Nd.

	**1**-Ce	**2**-Pr	**3**-Nd
Empirical formula	$C_{34}H_{42}CeF_7N_8O_7S_2$	$C_{34}H_{40}F_7N_8O_7PrS_2$	$C_{34}H_{42}F_7N_8NdO_7S_2$
Formula weight	1011.99	1010.77	1016.11
Temperature/K	150	150	150
Crystal system	orthorhombic	orthorhombic	orthorhombic
Space group	Pccn	Pccn	Pccn
a/Å	21.6177(18)	21.6076(8)	21.5899(9)
b/Å	11.3735(10)	11.3571(5)	11.3327(5)
c/Å	15.9181(12)	15.9279(6)	15.9150(5)
α/°	90	90	90
β/°	90	90	90
γ/°	90	90	90
Volume/Å^3	3913.8(6)	3908.7(3)	3894.0(3)
Z	4	4	4
ρ_{calc}g/cm^3	1.717	1.718	1.733
μ (MoKα) /mm^{-1}	1.362	1.445	1.533
F(000)	2044	2040	2052
Crystal size/mm^3	0.21 × 0.1 × 0.06	0.13 × 0.13 × 0.07	0.09 × 0.09 × 0.022
2Θ range /°	4.788 to 56.528	4.792 to 56.546	4.798 to 56.57
Index ranges	-28 ≤ h ≤ 26, -15 ≤ k ≤ 10, -20 ≤ l ≤ 21	-28 ≤ h ≤ 28, -15 ≤ k ≤ 14, -20 ≤ l ≤ 21	-28 ≤ h ≤ 28, -15 ≤ k ≤ 12, -20 ≤ l ≤ 21
Reflections collected	21771	38606	42892
Independent reflections	4849 [R_{int} = 0.0651, R_{sigma} = 0.0544]	4846 [R_{int} = 0.0395, R_{sigma} = 0.0248]	4836 [R_{int} = 0.0521, R_{sigma} = 0.0301]
Data/restraints /parameters	4849/0/272	4846/0/272	4836/1/272
Goodness-of-fit on F^2	1.03	1.103	1.047
Final R indexes [I>=2σ (I)]	R_1 = 0.0356, wR_2 = 0.0801	R_1 = 0.0264, wR_2 = 0.0617	R_1 = 0.0261, wR_2 = 0.0582
Final R indexes [all data]	R_1 = 0.0596, wR_2 = 0.1004	R_1 = 0.0412, wR_2 = 0.0855	R_1 = 0.0362, wR_2 = 0.0638
Largest diff. peak/hole / e Å^{-3}	0.83/-0.62	0.71/-0.49	0.79/-0.46

Table S2. Crystallographic information for **4**-Eu, **5**-Tb and **6**-Ho.

	**4**-Eu	**5**-Tb	**6**-Ho
Empirical formula	$C_{34}H_{42}EuF_7N_8O_7S_2$	$C_{34}H_{42}F_7N_8O_7S_2Tb$	$C_{34}H_{42}F_7HoN_8O_7S_2$
Formula weight	1023.83	1030.79	1036.8
Temperature/K	150	150	150
Crystal system	orthorhombic	orthorhombic	orthorhombic
Space group	$P2_12_12$	$P2_12_12$	$P2_12_12$
a/Å	11.3888(4)	11.3550(9)	11.3207(8)
b/Å	21.5973(8)	21.5456(13)	21.5056(13)
c/Å	7.7673(2)	7.7599(5)	7.7570(4)
α/°	90	90	90
β/°	90	90	90
γ/°	90	90	90
Volume/Å^3	1910.50(11)	1898.5(2)	1888.5(2)
Z	2	2	2
ρ_{calc}g/cm^3	1.78	1.803	1.823
μ (MoKα) /mm^{-1}	1.845	2.068	2.301
F(000)	1032	1036	1040
Crystal size/mm^3	0.25 × 0.08 × 0.08	0.13 × 0.07 × 0.07	0.1 × 0.09 × 0.05
2Θ range /°	5.198 to 56.578	5.212 to 56.648	5.224 to 56.638
Index ranges	$-15 \le h \le 15$, $-28 \le k \le 28$, $-9 \le l \le 10$	$-15 \le h \le 15$, $-28 \le k \le 28$, $-10 \le l \le 10$	$-11 \le h \le 15$, $-28 \le k \le 28$, $-10 \le l \le 9$
Reflections collected	20918	28405	13390
Independent reflections	4711 [R_{int} = 0.0654, R_{sigma} = 0.0527]	4700 [R_{int} = 0.1683, R_{sigma} = 0.1028]	4670 [R_{int} = 0.0700, R_{sigma} = 0.0783]
Data/restraints /parameters	4711/1/277	4700/0/273	4670/4/277
Goodness-of-fit on F^2	1.055	1.002	1.049
Final R indexes [I>=2σ (I)]	R_1 = 0.0266, wR_2 = 0.0661	R_1 = 0.0486, wR_2 = 0.1015	R_1 = 0.0389, wR_2 = 0.0909
Final R indexes [all data]	R_1 = 0.0277, wR_2 = 0.0671	R_1 = 0.0587, wR_2 = 0.1055	R_1 = 0.0456, wR_2 = 0.0948
Largest diff. peak/hole / e Å^{-3}	1.28/-1.36	1.29/-2.17	1.05/-1.25
Flack parameter	0.058(13)	0.38(2)	0.642(17)

Table S3. Crystallographic information for **7**-Er, **8**-Tm and **9**-Yb.

	**7**-Er	**8**-Tm	**9**-Yb
Empirical formula	$C_{34}H_{42}ErF_7N_8O_7S_2$	$C_{34}H_{42}F_7N_8O_7S_2Tm$	$C_{34}H_{42}F_7N_8O_7S_2Yb$
Formula weight	1039.13	1040.8	1044.91
Temperature/K	150	150	150
Crystal system	orthorhombic	orthorhombic	orthorhombic
Space group	$P2_12_12$	$P2_12_12$	$P2_12_12$
a/Å	11.3171(7)	11.2936(4)	11.2930(8)
b/Å	21.5211(13)	21.5207(9)	21.5051(13)
c/Å	7.7644(4)	7.7546(3)	7.7679(4)
α/°	90	90	90
β/°	90	90	90
γ/°	90	90	90
Volume/Å^3	1891.07(19)	1884.73(13)	1886.5(2)
Z	2	2	2
ρ_{calc}g/cm^3	1.825	1.834	1.84
μ (MoKα) /mm^{-1}	2.425	2.56	2.685
F(000)	1042	1044	1046
Crystal size/mm^3	0.17 × 0.07 × 0.04	0.17 × 0.16 × 0.11	0.13 × 0.1 × 0.06
2Θ range /°	5.224 to 56.598	5.228 to 56.57	5.232 to 56.618
Index ranges	$-15 \le h \le 14$, $-28 \le k \le 28$, $-10 \le l \le 9$	$-15 \le h \le 14$, $-28 \le k \le 28$, $-10 \le l \le 10$	$-15 \le h \le 15$, $-25 \le k \le 28$, $-9 \le l \le 10$
Reflections collected	22408	27568	20015
Independent reflections	4695 [R_{int} = 0.0651, R_{sigma} = 0.0519]	4641 [R_{int} = 0.0393, R_{sigma} = 0.0331]	4680 [R_{int} = 0.0660, R_{sigma} = 0.0549]
Data/restraints /parameters	4695/1/276	4641/3/276	4680/2/281
Goodness-of-fit on F^2	1.024	1.089	1.103
Final R indexes [I>=2σ (I)]	R_1 = 0.0252, wR_2 = 0.0582	R_1 = 0.0178, wR_2 = 0.0400	R_1 = 0.0291, wR_2 = 0.0811
Final R indexes [all data]	R_1 = 0.0280, wR_2 = 0.0596	R_1 = 0.0184, wR_2 = 0.0402	R_1 = 0.0309, wR_2 = 0.0822
Largest diff. peak/hole / e Å^{-3}	0.60/-0.80	0.56/-1.15	1.73/-1.42
Flack parameter	0.016(7)	0.012(4)	0.046(13)

Table S4. Selected bond lengths for all analogues.

Atom	Atom	Length (Å)				
		Ce	Pr	Nd	Eu	Tb
Ln	F	2.206(3)	2.1921(19)	2.195(2)	2.160(2)	2.141(5)
Ln	N1	2.748(3)	2.737(2)	2.721(2)	2.694(3)	2.686(7)
Ln	N2	2.767(3)	2.757(2)	2.741(2)	2.705(3)	2.693(7)
Ln	N3	2.669(3)	2.649(2)	2.6368(19)	2.577(3)	2.550(8)
Ln	N4	2.680(3)	2.665(2)	2.647(2)	2.570(3)	2.544(8)
Atom	Atom	Length (Å)				
		Ho	Er	Tm	Yb	
Ln	F	2.130(5)	2.125(3)	2.1131(19)	2.096(3)	
Ln	N1	2.661(8)	2.664(4)	2.658(2)	2.659(4)	
Ln	N2	2.679(7)	2.672(3)	2.669(2)	2.654(5)	
Ln	N3	2.526(8)	2.527(4)	2.514(3)	2.492(5)	
Ln	N4	2.522(8)	2.507(4)	2.501(3)	2.507(5)	

Table S5. Selected bond angles for complexes **1**-Ce, **2**-Pr, **3**-Nd, **4**-Eu.

**Atom**	**Atom**	**Atom**	**Angle / °**			
			Ce	Pr	Nd	Eu
F1	Nd1	N1	130.23(6)	130.13(4)	129.94(5)	128.28(7)
F1	Nd1	N2	130.25(6)	130.17(5)	129.85(5)	128.58(7)
F1	Nd1	N3	73.21(6)	73.27(5)	72.61(4)	73.83(8)
F1	Nd1	N4	73.43(6)	72.96(5)	72.89(4)	73.63(8)
N1	Nd1	N2	65.15(9)	65.58(6)	65.60(6)	67.27(9)
N1	Nd1	N2[1]	65.52(9)	65.28(6)	65.81(6)	67.27(9)
N1[1]	Nd1	N1	99.50(12)	99.65(9)	100.11(10)	103.44(14)
N1[1]	Nd1	N2[1]	65.15(9)	65.28(6)	65.60(6)	67.27(9)
N2[1]	Nd1	N2	99.54(12)	99.73(9)	100.29(10)	102.84(14)
N3	Nd1	N1	84.14(9)	84.03(7)	84.07(6)	74.27(10)
N3	Nd1	N1[1]	118.39(9)	118.82(7)	119.15(6)	128.04(10)
N3	Nd1	N2	60.90(9)	61.19(6)	61.34(6)	64.31(10)
N3	Nd1	N2[1]	149.26(9)	149.28(6)	149.62(6)	141.33(10)
N3	Nd1	N4	85.97(10)	85.83(7)	85.39(6)	86.65(11)
N3	Nd1	N4[1]	84.58(10)	84.49(7)	84.52(6)	84.35(11)
N3[1]	Nd1	N2	149.25(9)	149.28(6)	149.61(6)	141.33(10)
N3[1]	Nd1	N2[1]	60.90(9)	61.19(6)	61.34(6)	64.32(10)
N3[1]	Nd1	N3	146.41(12)	145.92(9)	145.23(8)	147.67(15)
N3[1]	Nd1	N4[1]	85.97(10)	85.83(7)	85.39(6)	86.65(11)
N4	Nd1	N1	61.06(9)	61.27(7)	61.38(7)	64.87(11)
N4	Nd1	N1[1]	148.47(9)	148.45(7)	148.97(6)	140.69(10)
N4	Nd1	N2	118.61(9)	119.02(7)	119.23(6)	129.06(10)
N4	Nd1	N2[1]	83.66(9)	83.46(7)	83.62(6)	73.82(10)
N4	Nd1	N4[1]	146.87(13)	146.53(10)	145.77(8)	147.27(16)
N4[1]	Nd1	N1[1]	61.06(9)	61.27(7)	61.38(7)	64.87(11)
N4[1]	Nd1	N2[1]	118.61(9)	119.02(7)	119.23(6)	129.06(10)

Table S6. Selected bond angles for the complexes **5**-Tb, **6**-Ho, **7**-Er, **8**-Tm, **9**-Yb.

**Atom**	**Atom**	**Atom**	**Angle / °**				
			Tb	Ho	Er	Tm	Yb
F1	Nd1	N1	128.15(15)	128.17(16)	128.07(8)	127.97(6)	128.31(10)
F1	Nd1	N2	128.49(15)	128.41(16)	128.50(8)	128.38(5)	128.03(10)
F1	Nd1	N3	73.50(15)	73.19(18)	73.40(9)	73.30(6)	72.97(11)
F1	Nd1	N4	73.51(16)	72.78(18)	73.14(9)	73.01(6)	73.34(11)
N1	Nd1	N2	67.3(2)	67.4(2)	67.47(10)	67.59(7)	67.51(13)
N1	Nd1	N2[1]	67.4(2)	67.4(2)	67.39(10)	67.49(7)	67.58(13)
N1[1]	Nd1	N1	103.7(3)	103.7(3)	103.87(16)	104.06(11)	103.38(19)
N2[1]	Nd1	N2	103.0(3)	103.2(3)	103.01(15)	103.23(11)	103.9(2)
N3	Nd1	N1	65.0(2)	74.3(2)	73.91(11)	73.90(8)	73.60(14)
N3	Nd1	N1[1]	140.6(2)	129.0(2)	129.00(11)	129.11(8)	130.20(14)
N3	Nd1	N2	73.6(2)	65.0(2)	65.02(11)	65.11(8)	65.67(14)
N3	Nd1	N2[1]	129.4(2)	141.4(2)	141.07(11)	141.05(8)	140.60(14)
N3	Nd1	N4	84.5(3)	83.9(2)	84.33(12)	86.26(9)	84.29(16)
N3	Nd1	N4[1]	86.2(3)	86.3(2)	86.17(12)	84.10(9)	86.08(16)
N3[1]	Nd1	N1	140.6(2)	129.0(2)	129.00(11)	129.11(8)	130.20(14)
N3[1]	Nd1	N1[1]	65.0(2)	74.3(2)	73.91(11)	73.90(8)	73.60(14)
N3[1]	Nd1	N3	147.0(3)	146.4(4)	146.81(17)	146.61(12)	145.9(2)
N3[1]	Nd1	N4[1]	84.5(3)	83.9(2)	84.33(12)	86.26(9)	84.29(16)
N4	Nd1	N1	74.1(2)	65.9(2)	65.45(12)	65.60(8)	65.11(15)
N4	Nd1	N1[1]	128.7(2)	140.8(2)	140.64(11)	140.60(8)	140.93(14)
N4	Nd1	N2	141.1(2)	130.1(2)	129.81(11)	130.19(8)	129.24(15)
N4	Nd1	N2[1]	64.8(2)	73.9(2)	73.79(11)	73.60(8)	73.78(14)
N4	Nd1	N4[1]	147.0(3)	145.6(4)	146.28(18)	146.01(13)	146.7(2)
N4[1]	Nd1	N1	128.7(2)	140.8(2)	140.64(11)	140.60(8)	140.93(14)
N4[1]	Nd1	N1[1]	74.1(2)	65.9(2)	65.45(12)	65.60(8)	65.11(15)
N4[1]	Nd1	N2[1]	141.1(2)	73.9(2)	73.79(11)	73.60(8)	73.78(14)

Table S7. SHAPE analysis results for complexes **1**-Ce to **9**-Yb. Bold font highlights the lowest value, which corresponds to the least distorted symmetry.

**Molecular geometry shapes**	**1**-Ce	**2**-Pr	**3**-Nd	**4**-Eu	**5**-Tb	**6**-Ho	**7**-Er	**8**-Tm	**9**-Yb
Enneagon	37.63	37.59	37.63	37.28	37.30	37.53	37.46	37.28	37.40
Octagonal bipyramid	23.99	23.98	23.97	20.44	20.46	20.61	20.62	20.46	20.62
Heptagonal bipyramid	16.56	16.60	16.58	19.08	19.24	19.23	19.16	19.12	19.24
Triangular cupola	17.69	17.65	17.63	17.66	17.73	17.65	17.63	17.60	17.66
Capped cube (elongated square pyramid)	5.91	5.95	5.89	9.59	9.68	9.62	9.64	9.65	9.80
Capped cube	3.24	3.31	3.31	7.25	7.40	7.44	7.43	7.44	7.58
Capped sq. antiprism (gyroelongated sq. pyramid)	4.48	4.37	4.30	2.49	2.41	2.28	2.33	2.36	2.34
**Capped square antiprism**	**2.51**	**2.42**	**2.40**	**0.61**	**0.57**	**0.52**	**0.54**	**0.58**	**0.53**
Tricapped trigonal prism	5.93	5.83	5.77	4.06	3.99	3.87	3.92	3.95	3.94
Spherical tricapped trigonal prism	3.58	3.51	3.51	1.50	1.50	1.45	1.46	1.52	1.46
Tridiminished icosahedron	12.63	12.62	12.65	12.57	12.63	12.71	12.70	12.73	12.76
Hula-hoop	9.29	9.36	9.38	12.39	12.51	12.51	12.55	12.56	12.65
Muffin	3.21	3.14	3.12	1.45	1.42	1.37	1.39	1.45	1.39

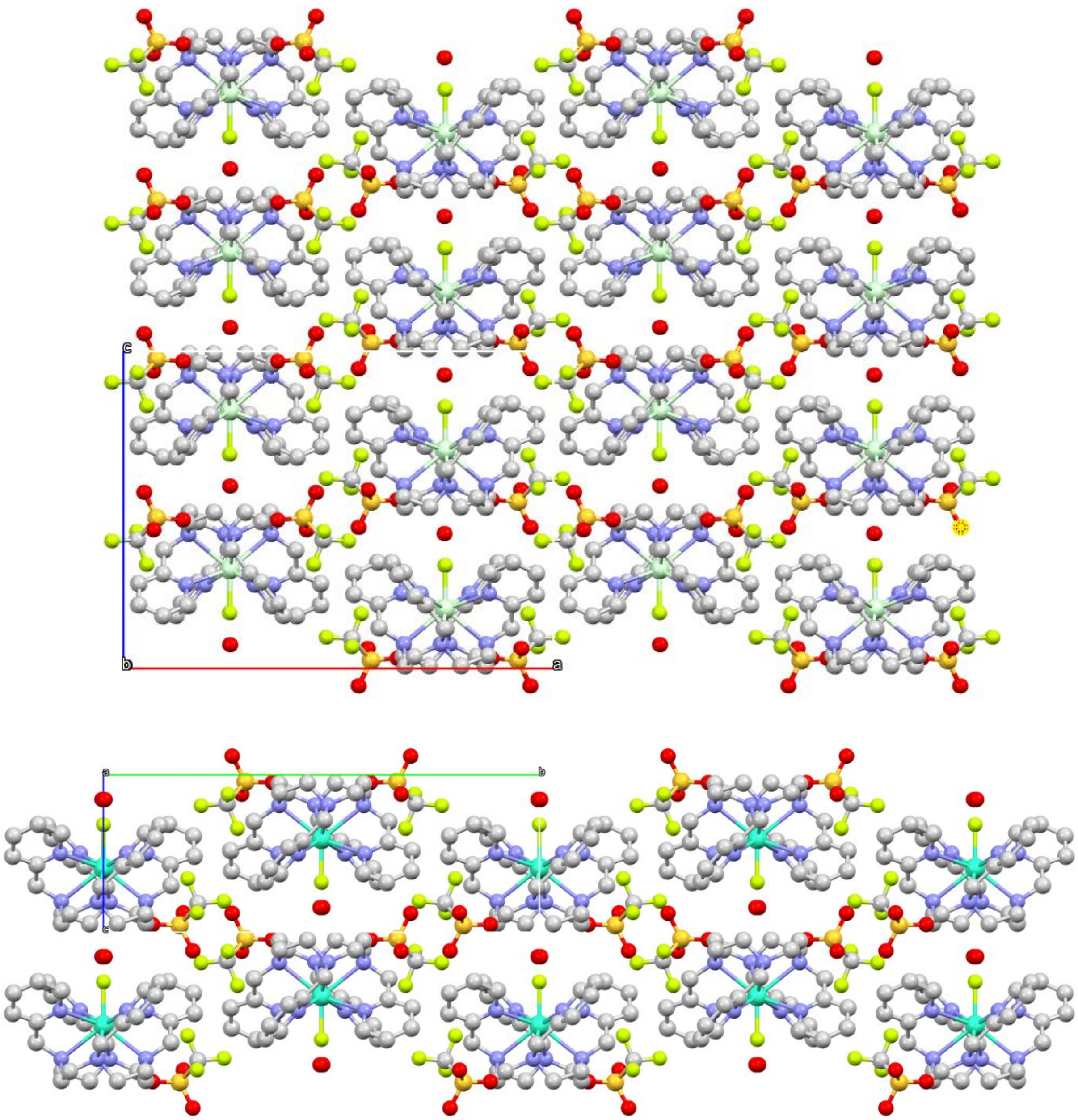

Figure S3. Representative examples of the crystal packing in Pccn (**3**-Nd, upper) and $P2_12_12$ (**5**-Tb, lower). The *c*-axis is parallel to the Ln-F bond. C, grey; F, lime green; N, blue; Nd, pale green, O, red: S, Yellow; Tb, cyan. H omitted for clarity.

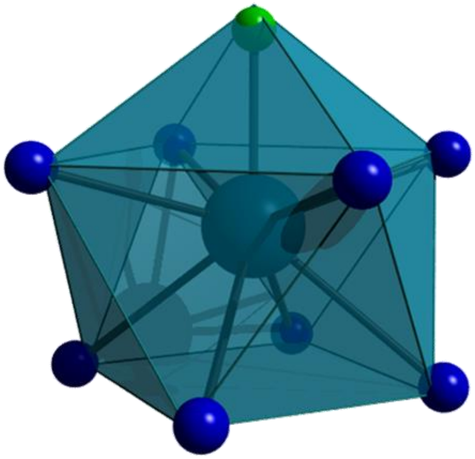

Figure S4. Representation of the ideal capped squared antiprism with the first coordination sphere to show the distortion of the geometry.

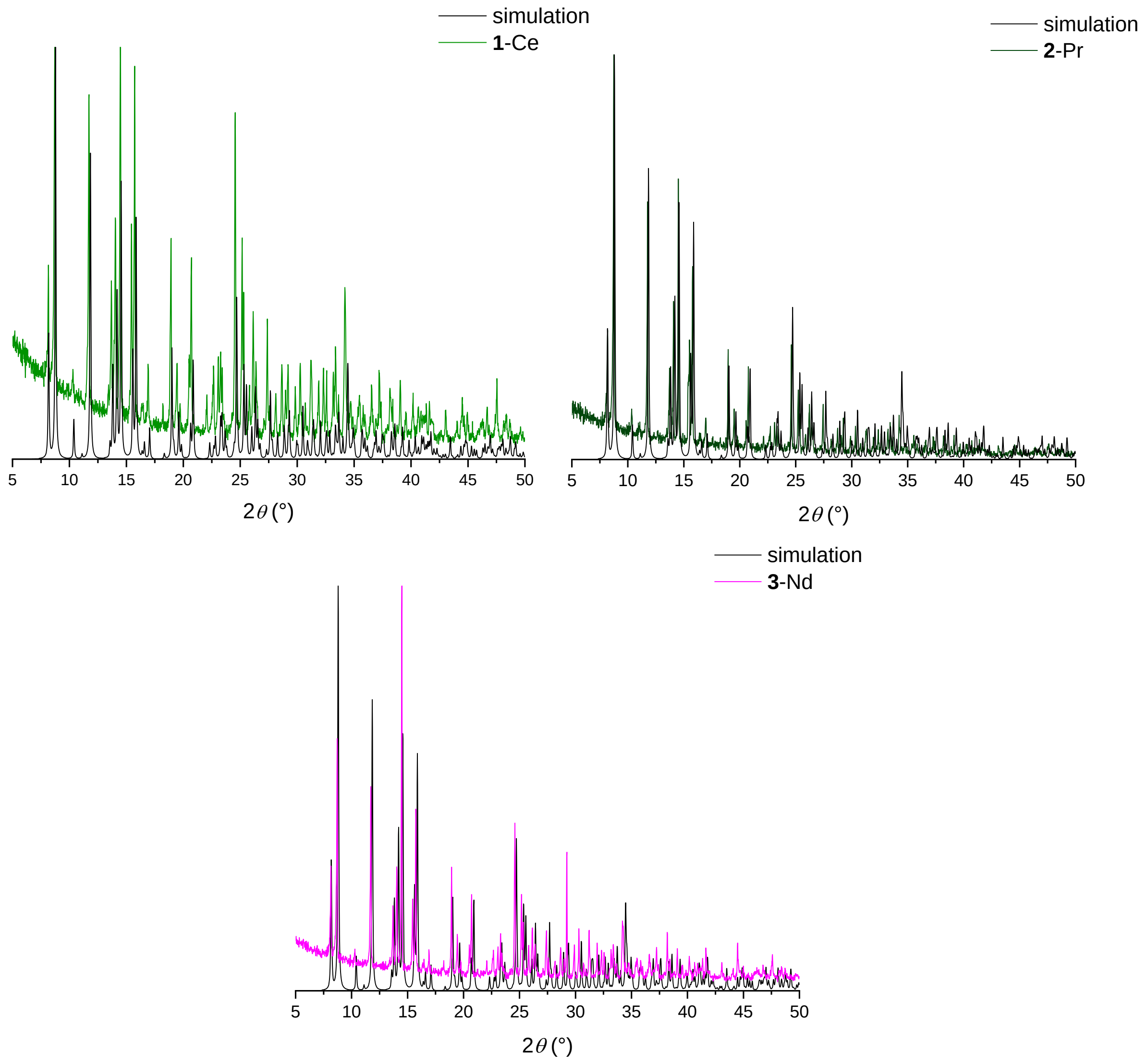

Figure S5. The PXRD pattern of the analogues that crystallize in the Pccn space group, **1**-Ce, **2**-Pr and **3**-Nd.

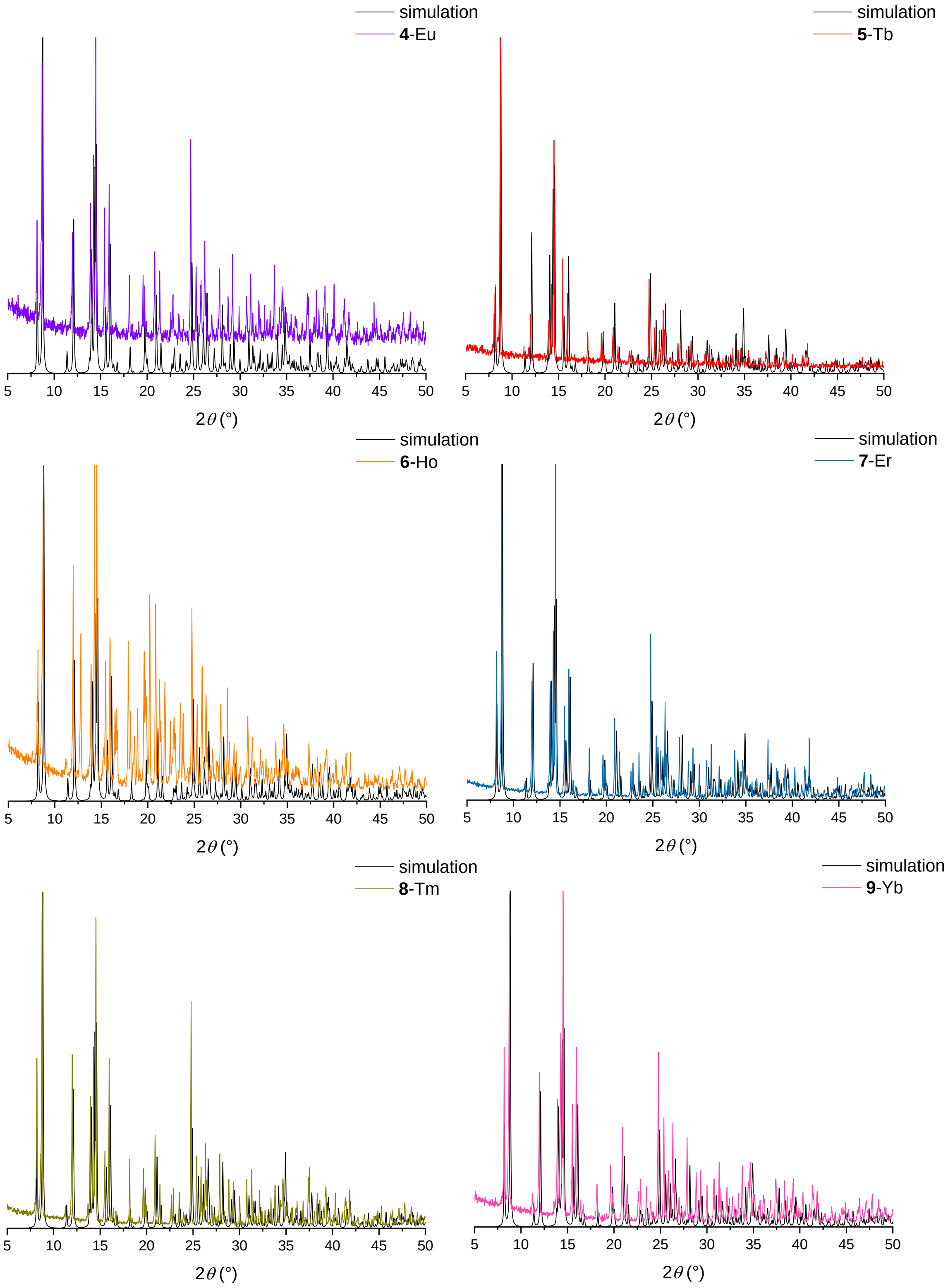

Figure S6. The PXRD pattern of the analogues that crystallize in the group $P2_12_12$ space group.

3. Magnetic characterization

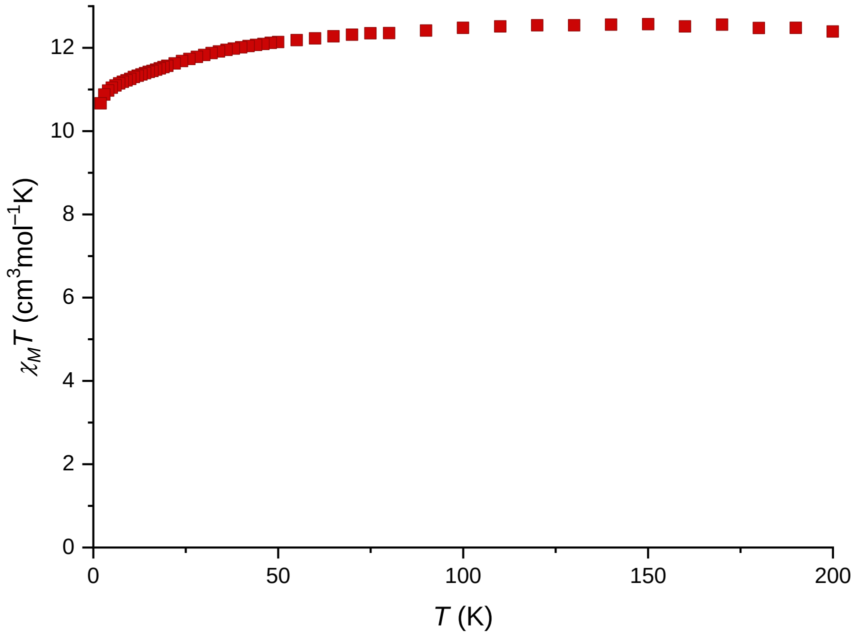

Figure S7. Temperature dependence of $\chi_M T$ from 290-2 K of the diluted sample **12**-Tb@Y.

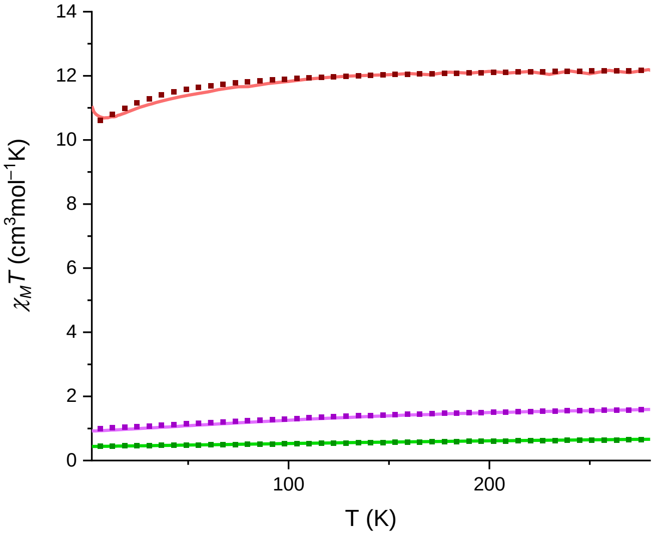

Figure S8. *Ab initio* calculated and experimental values of χ_MT for **1**-Ce (green), **3**-Nd (purple) and **5**-Tb (red).

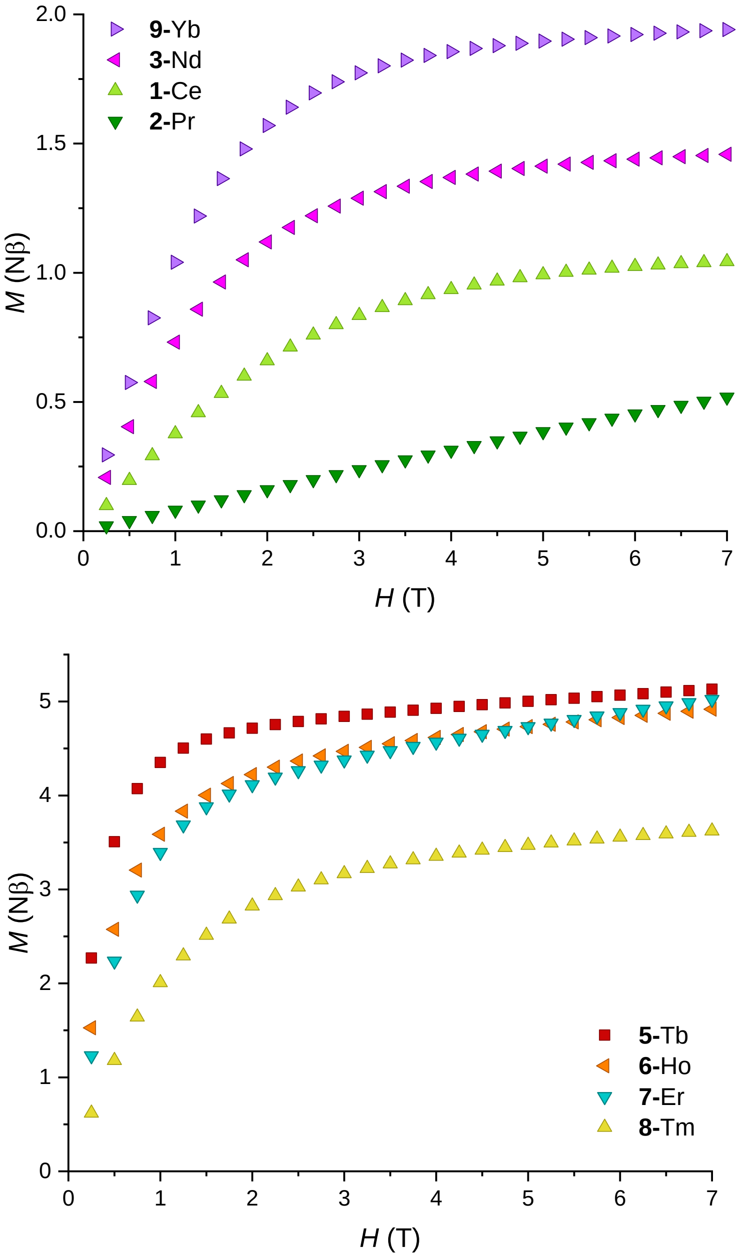

Figure S9. Magnetization of complexes **1**-Ce to **9**-Yb (excluding **4**-Eu) at 2 K.

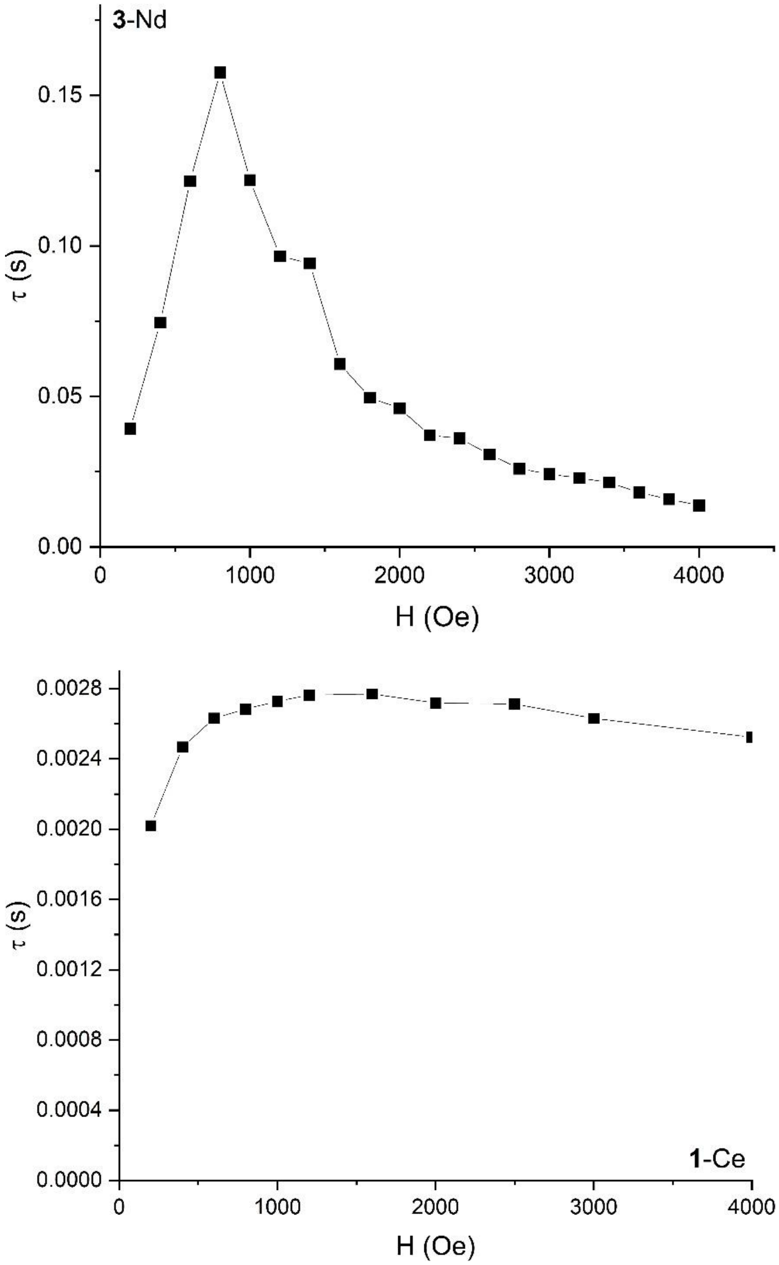

Figure S10. Relaxation times (τ) as a function of the applied field (Oe) for **1**-Ce (upper) and **3**-Nd (lower), showing the optimum dc field as 1200 Oe and 800 Oe, respectively. Solid lines are a guide for the eye.

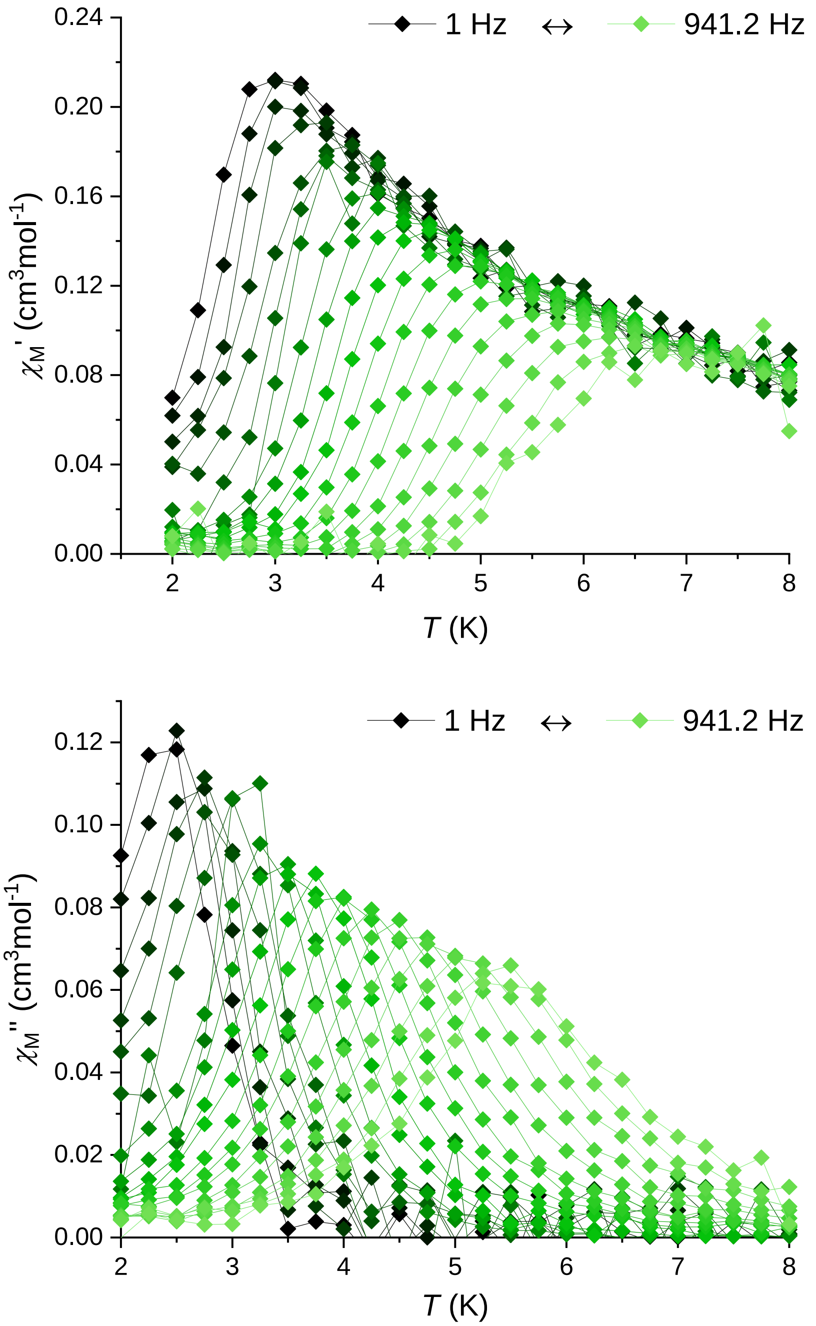

Figure S11. Temperature dependence of the in-phase, χ'_M (upper), and out-of-phase, χ''_M (lower) ac susceptibility, in a 1200 Oe dc field, for **1**-Ce with ac frequencies between 1−941.2 Hz. Solid lines are a guide for the eye.

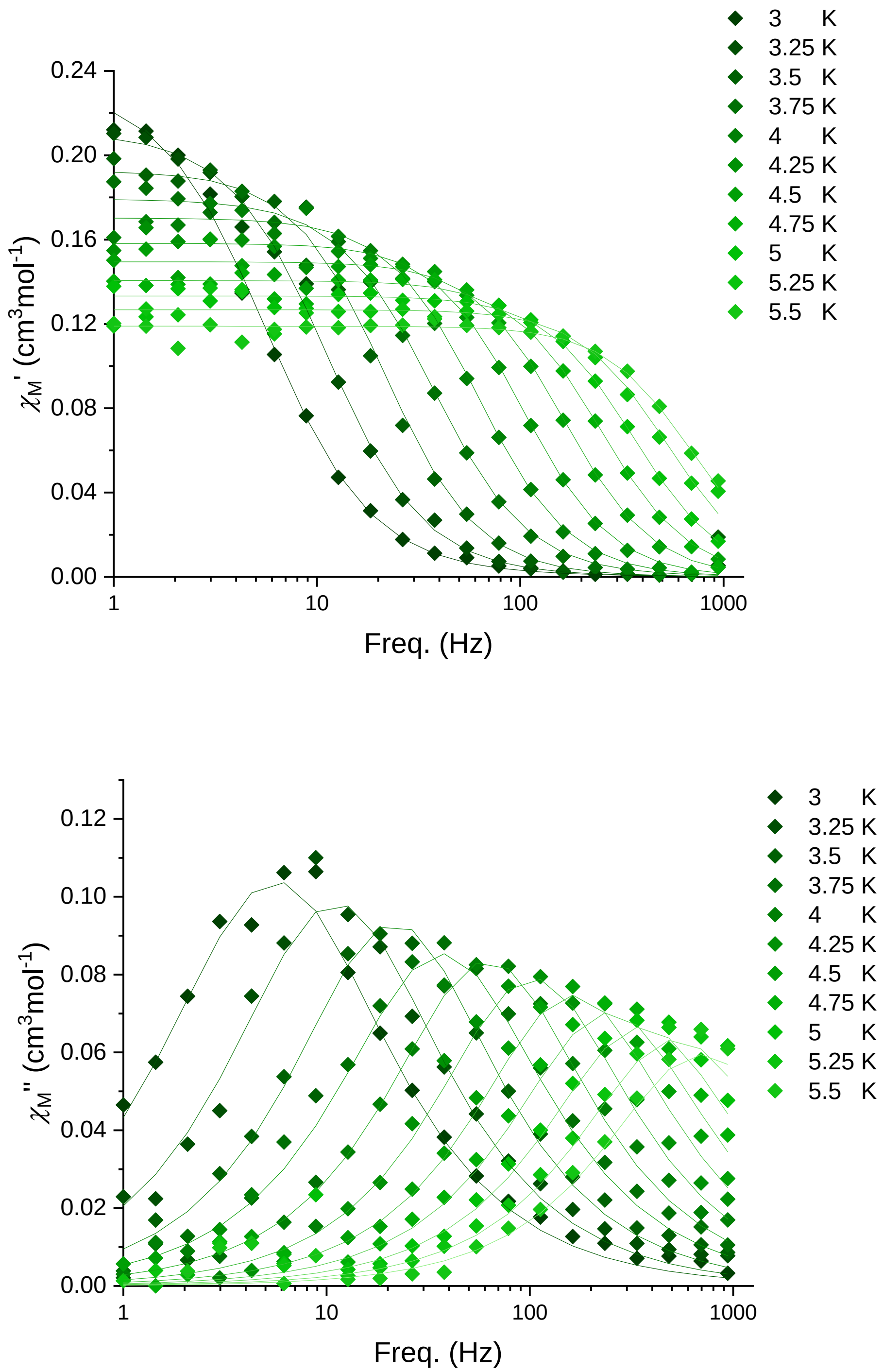

Figure S12. Frequency dependence of the in-phase, χ'_M (upper), and out-of-phase, χ''_M (lower) ac susceptibility, in a 1200 Oe dc field, for **1**-Ce. Solid lines correspond to the best fit.

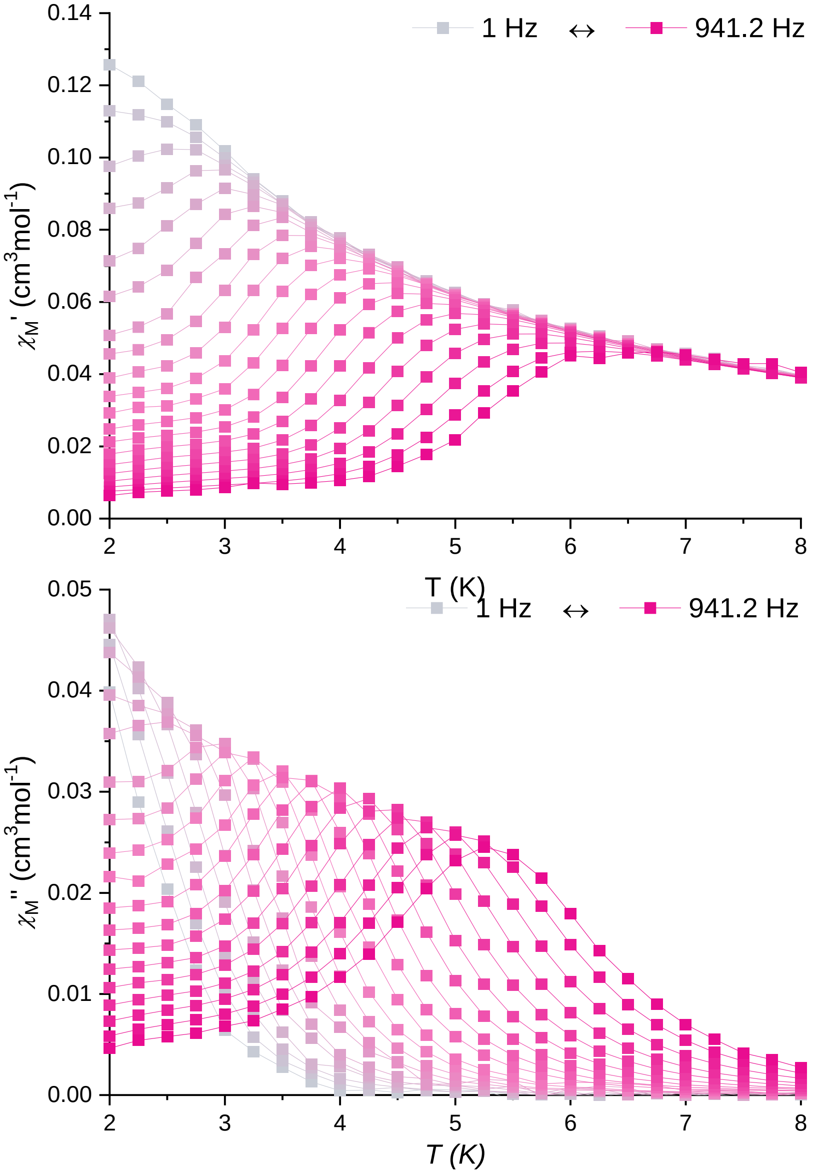

Figure S13. Temperature dependence of the in-phase, χ'_M (upper), and out-of-phase, χ''_M (lower) ac susceptibility, in a 800 Oe dc field, for **3**-Nd with ac frequencies between 1−941.2 Hz. Solid lines are a guide for the eye.

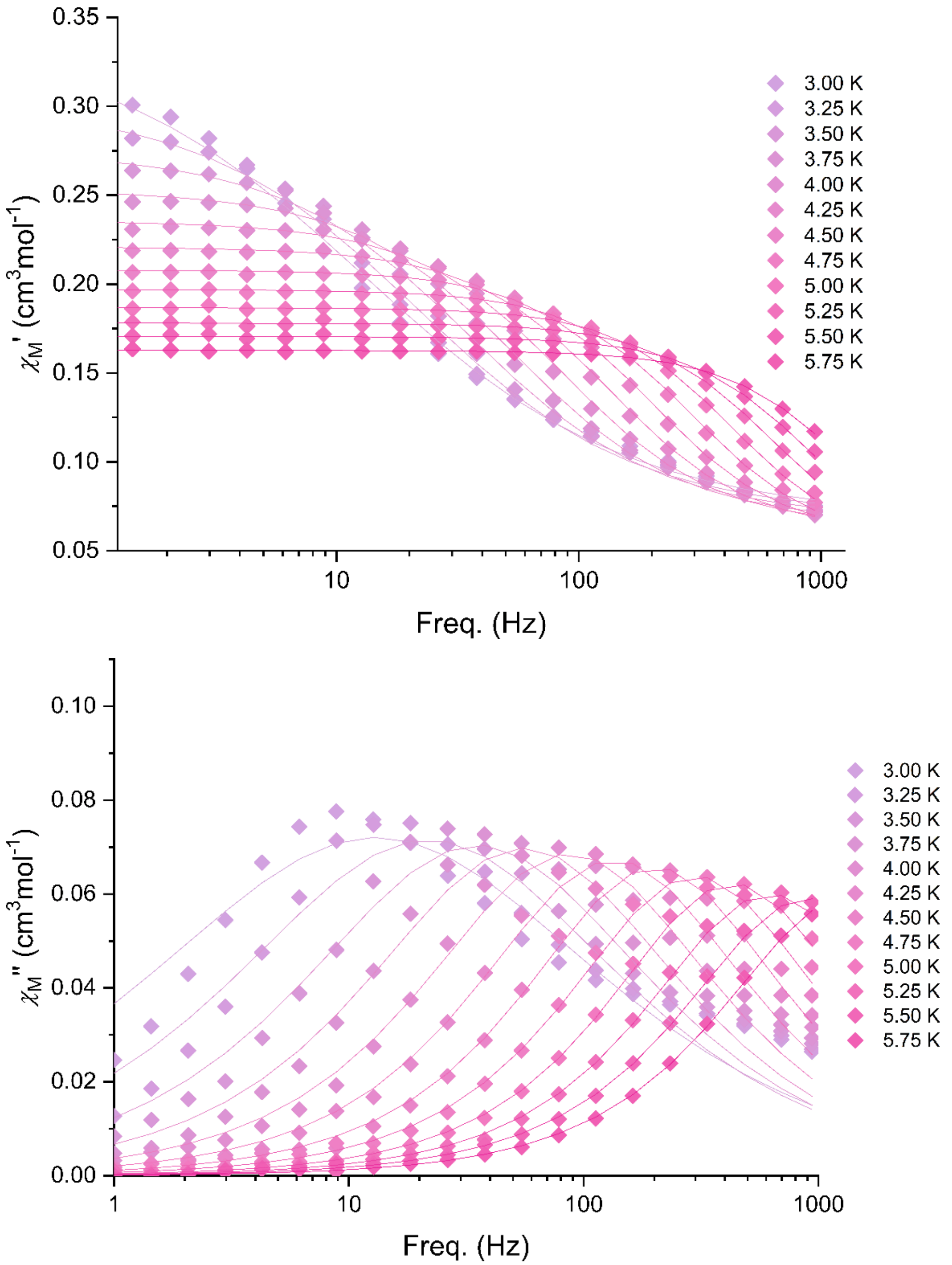

Figure S14. Frequency dependence of the in-phase, χ'_M (upper), and out-of-phase, χ''_M (lower) ac susceptibility, in a 800 Oe dc field, for **3**-Nd. Solid lines correspond to the best fit.

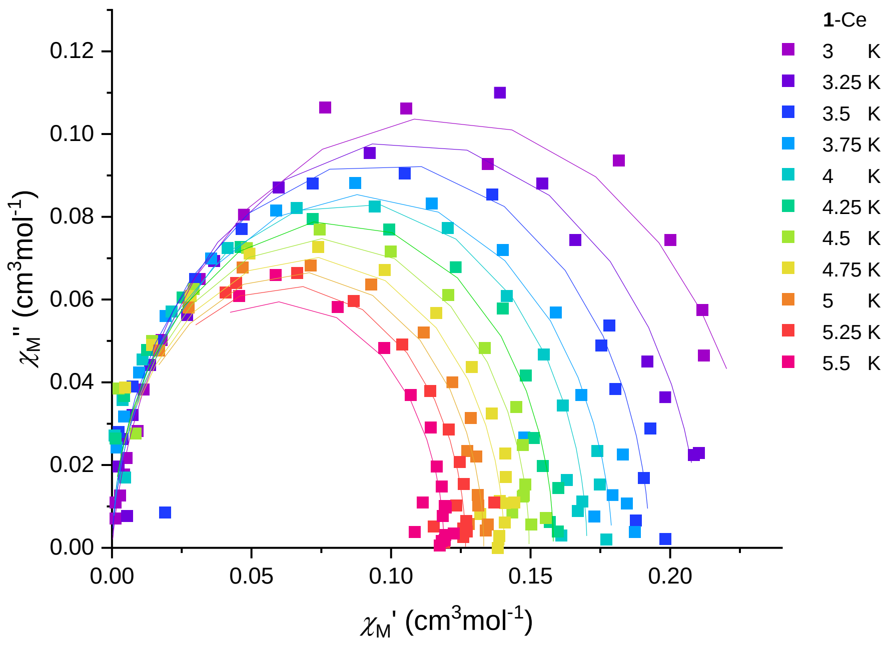

Figure S15. χ''_M *vs.* χ'_M plot of the ac magnetic susceptibility of **1**-Ce, in a 1200 Oe dc field. Solid lines correspond to the best fit to Debye's law.

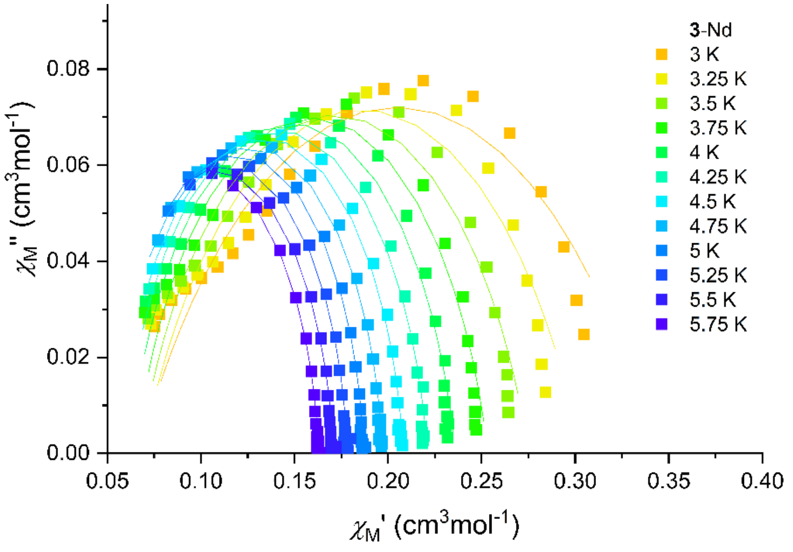

Figure S16. χ''_M *vs.* χ'_M plot of the ac magnetic susceptibility of **3**-Nd, in a 800 Oe dc field. Solid lines correspond to the best fit to Debye's law.

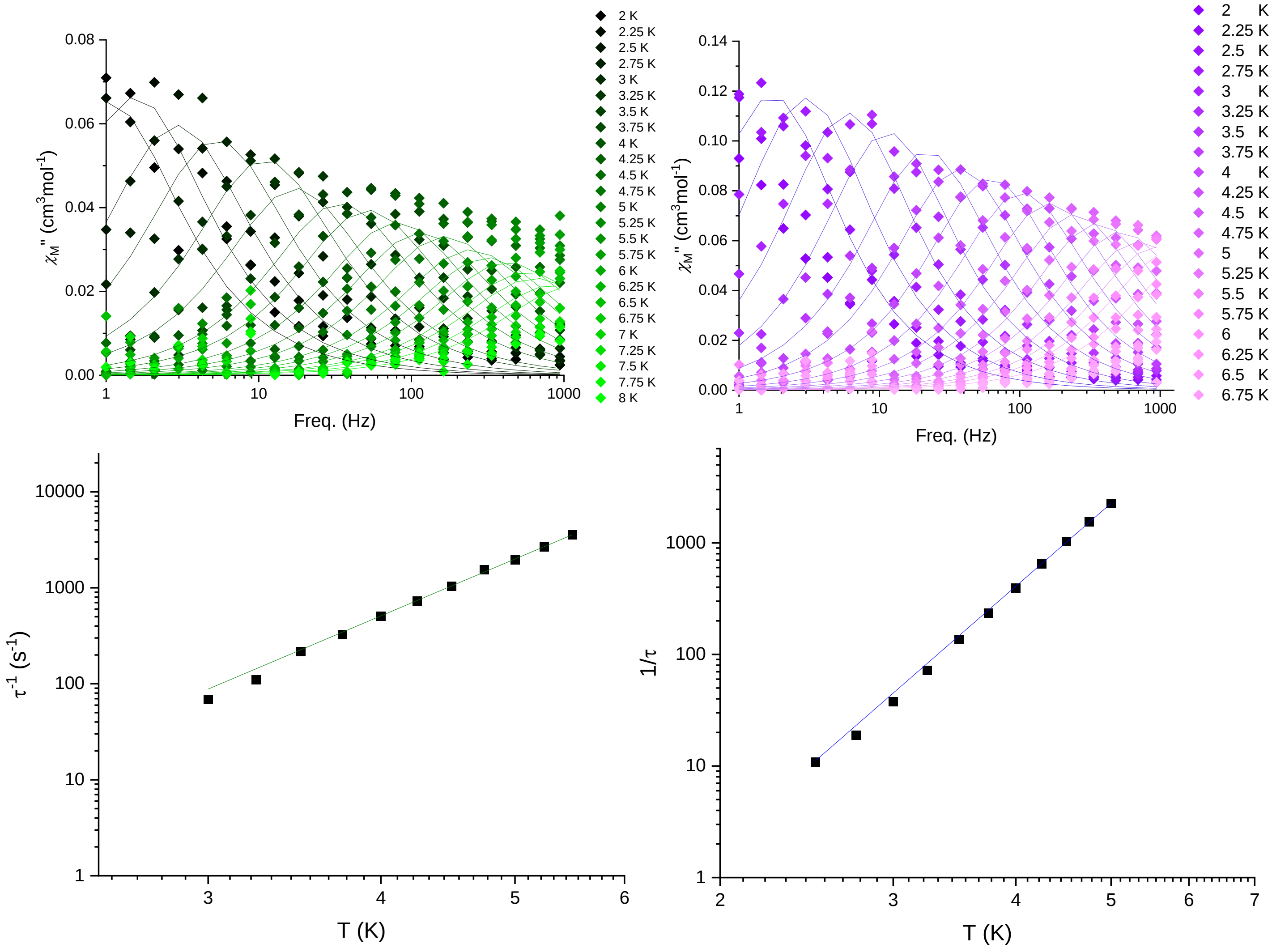

Figure S17. (top) Out-of-phase ac susceptibility *vs.* frequency measured at 1400 and 1200 Oe for **10**-Ce@La (left) and **11**-Nd@La (right), respectively. (bottom) Dependence of 1/τ *vs.* T for **10**-Ce@La (left) and **11**-Nd@La (right), measured at 1400 Oe and 1200 Oe, respectively. Best fit for **10**-Ce@La: C = 0.107 K^{-n} s^{-1}, n = 6.1. Best fit for **11**-Nd@La: C = 0.0098 K^{-n} s^{-1}, n = 7.7.

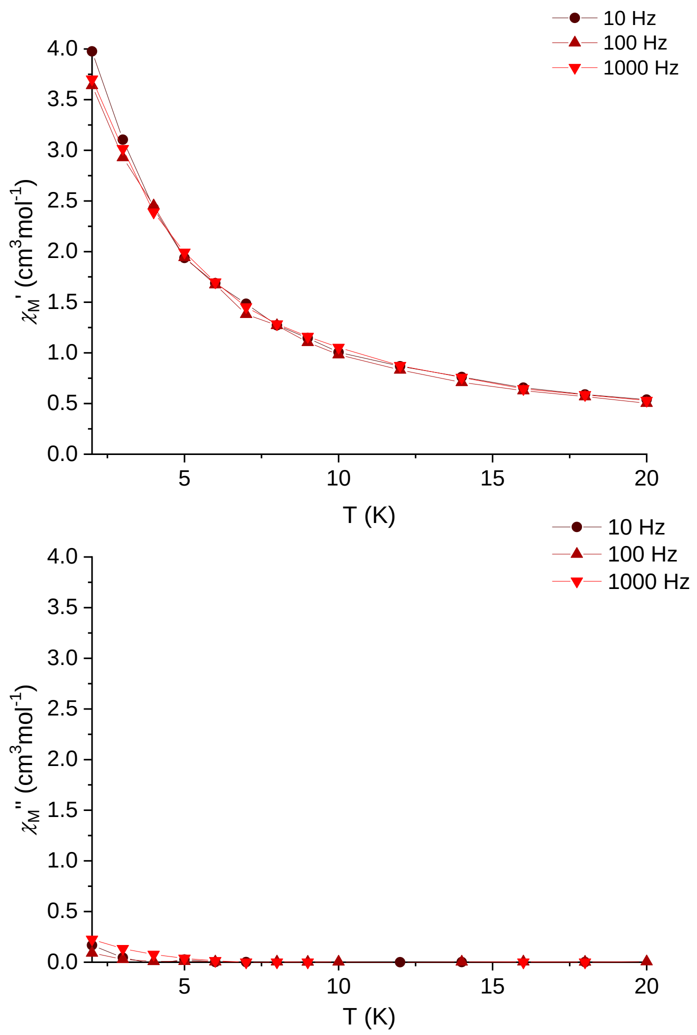

Figure S18. In-phase and out-of-phase ac susceptibility of the diluted sample **12**Tb@Y measured in a 2000 Oe dc field.

3. Ab initio calculations

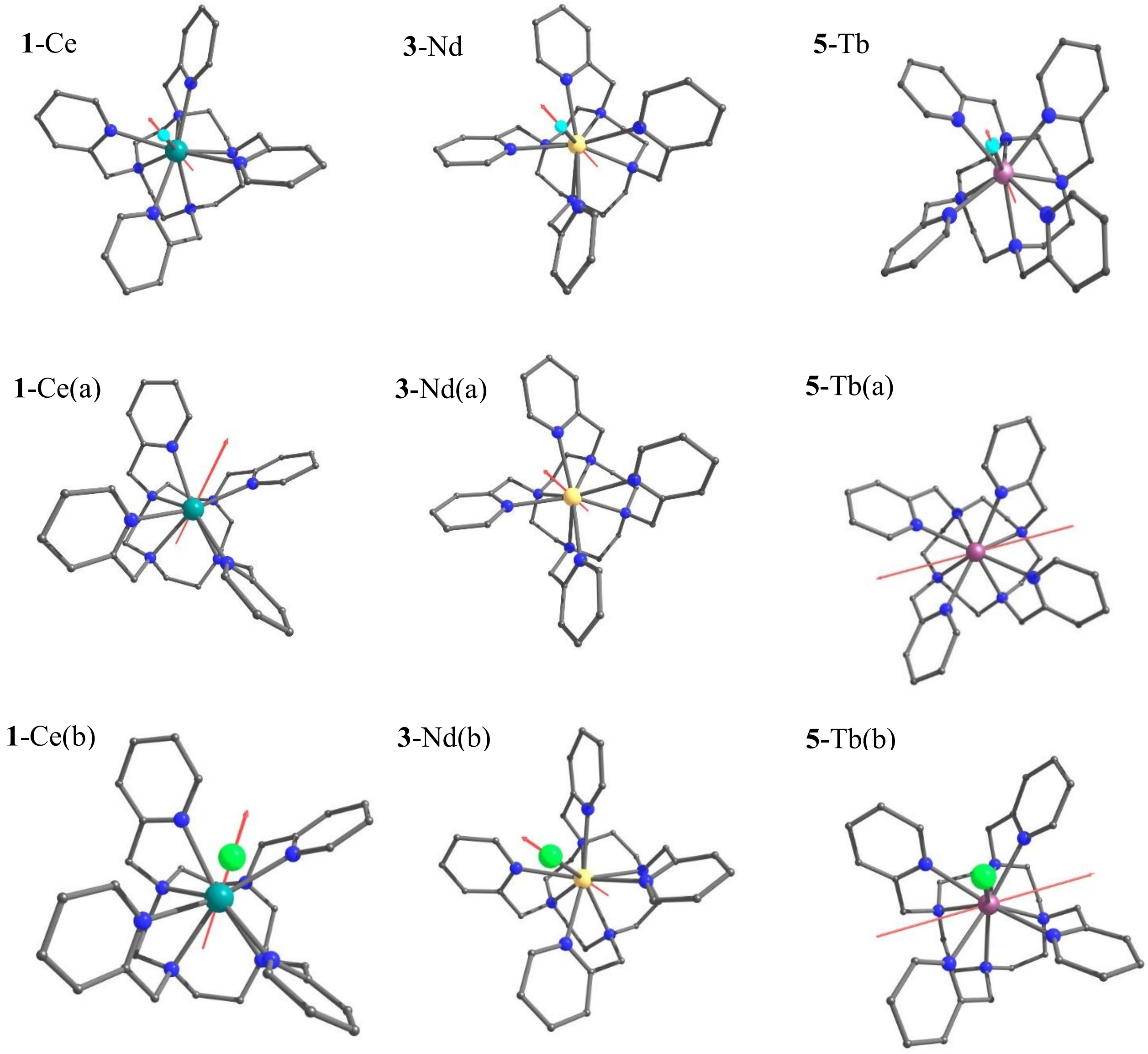

Figure S19. The anisotropy axis of the ground KD (red arrow). *Row 1:* **1**-Ce(left), **3**-Nd (middle) and **5**-Tb(right). *Row 2:* Ln(a) are computational models where the axial F^- ligand is removed, leaving an $\{LnN_8\}$ coordination environment. *Row 3:* Ln(b) are computational models where the axial F^- ligand is replaced by an I^- ligand. The Ln(a) and Ln(b) models have been optimized. Ce, cyan; Nd, yellow; Tb, pink; F, light blue; I, green; N, dark blue; C, grey; H omitted for clarity.

Table S8. LoProp charges of the atoms in **1**-Ce, **3**-Nd and **5**-Tb and the computational models (Ln(a) are models where the axial F^{-} ligand is removed, leaving an $\{LnN_8\}$ coordination environment; Ln(b) are models where the axial F^{-} ligand is replaced by an I^{-} ligand).

	**1**-Ce	**3**-Nd	**5**-Tb
Ln	2.366	2.381	2.379
F	-0.881	-0.888	-0.894
N1	-0.409	-0.412	-0.4176
N3	-0.408	-0.4115	-0.4110
N5	-0.409	-0.4121	-0.4176
N7	-0.408	-0.4115	-0.4110
N2	-0.344	-0.3482	-0.3466
N4	-0.342	-0.3454	-0.3509
N6	-0.343	-0.3482	-0.3466
N8	-0.342	-0.3454	-0.3509

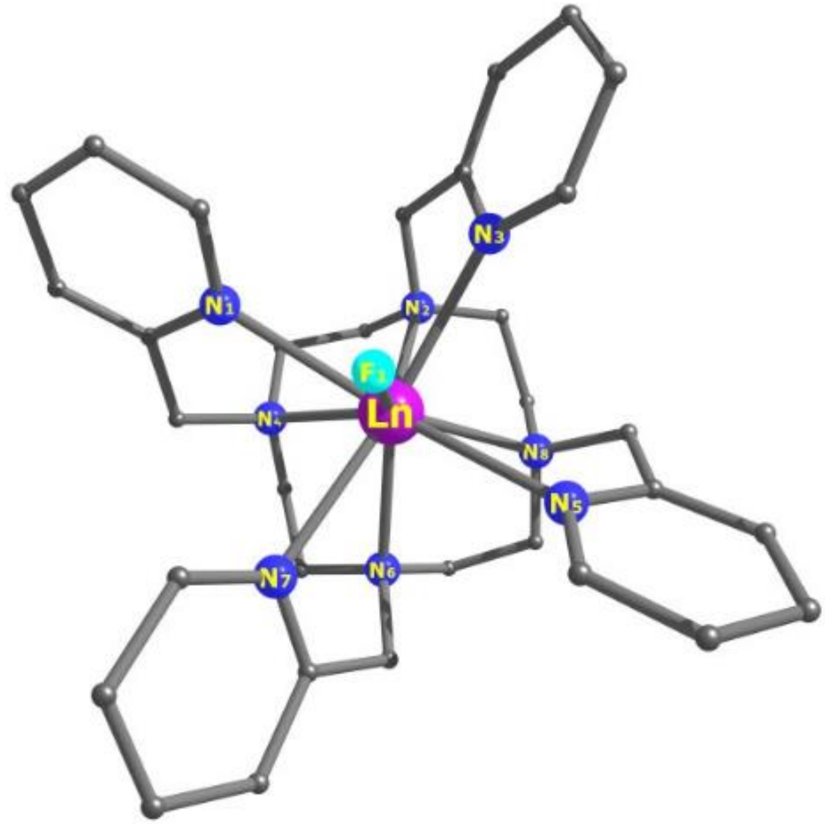

Table S8. Contd.

	**1**-Ce(a)	**3**-Nd(a)	**5**-Tb(a)
Ln	2.3615	2.363	2.3045
N1	-0.4570	-0.4594	-0.4611
N3	-0.4556	-0.4600	-0.4686
N5	-0.4570	-0.4594	-0.4611
N7	-0.4556	-0.4600	-0.4686
N2	-0.3580	-0.3621	-0.3606
N4	-0.3571	-0.3594	-0.3648
N8	-0.3580	-0.3621	-0.3606
N6	-0.3571	-0.3594	-0.3648

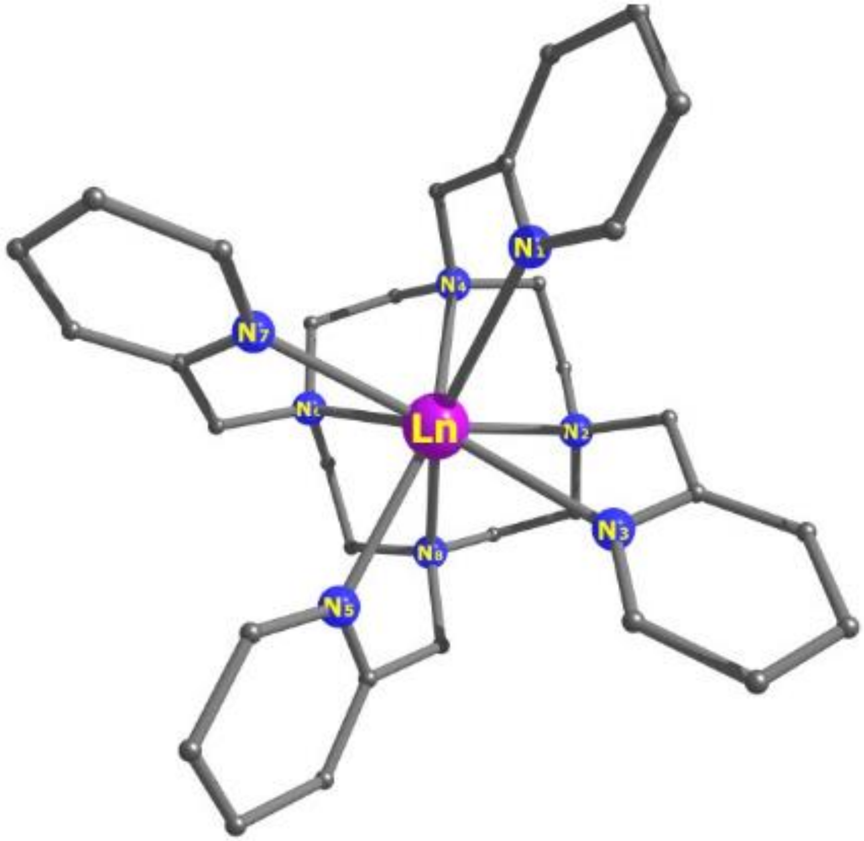

Table S8. Contd.

	**1**-Ce(b)	**3**-Nd(b)	**5**-Tb(b)
Ln	2.3022	2.3045	2.2951
I	-0.8015	-0.8005	-0.7996
N1	-0.4383	-0.4406	-0.4405
N3	-0.4383	-0.4405	-0.4405
N5	-0.4383	-0.4406	-0.4405
N7	-0.4383	-0.4406	-0.4405
N2	-0.3413	-0.3425	-0.3426
N4	-0.3413	-0.3424	-0.3426
N8	-0.3413	-0.3425	-0.3426
N6	-0.3413	-0.3425	-0.3426

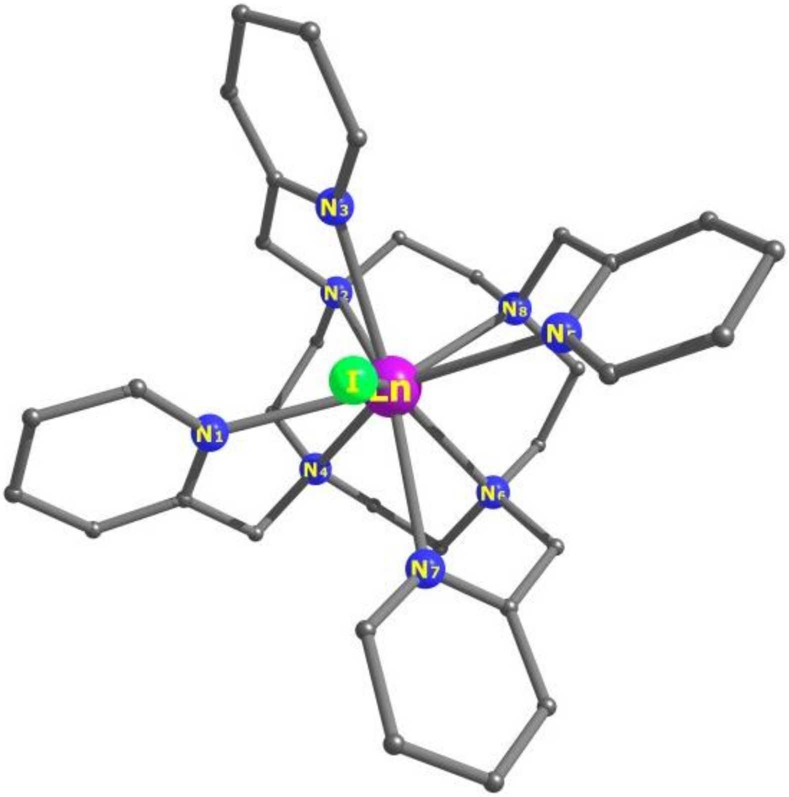

Table S9. *Ab initio* SINGLE_ANISO computed crystal field parameters for complexes **1**-Ce and **3**-Nd showing that **1**-Ce has larger crystal field than **3**-Nd. Here, k - the rank of the ITO, = 2, 4. q - the component of the ITO, = -k, -k+1, ... 0, 1, ... k; the non-axial term (B_k^q, where q ≠ 0 and k = 2, 4) to the axial term (B_k^q, where q = 0 and k = 2, 4) ratio should be smaller for lower QTM.

k	q	Complexes B_k^q	
		**1**-Ce	**3**-Nd
2	-2	-3.04	6.4×10^{-02}
2	-1	-1.69×10^{-01}	2.8×10^{-01}
**2**	**0**	**-30.0**	**-2.6**
2	1	-2.88	1.5×10^{-01}
2	2	1.83	-2.1×10^{-01}
4	-4	3.65	3.9×10^{-02}
4	-3	6.84×10^{-02}	-8.5×10^{-03}
4	-2	-1.06×10^{-01}	-8.4×10^{-04}
4	-1	-1.19×10^{-03}	4.9×10^{-03}
4	0	1.46×10^{-01}	-4.9×10^{-03}
4	1	-0.35×10^{-02}	1.8×10^{-03}
4	2	5.29×10^{-02}	1.6×10^{-03}
4	3	-2.66×10^{-01}	-4.6×10^{-03}
4	4	-2.75	8.1×10^{-02}

Table S10. Energies of states and tunnel splitting in **5**-Tb, **5**-Tb(a) and **5**-Tb(b). Model (a) has the axial F^{-} ligand removed and model (b) has the axial F^{-} replaced by I^{-}.

	**Energy (K)**	Δ_{tun}
**5**-Tb	0.0/0.6	0.6
	111.5/113.6	2.1
	221.6/242.4	20.8
	341.9/361.2	19.3
	423.3/567.9	144.6
	598.5/621.7	23.2
**5**-Tb(a)	0.0/8.7	8.7
	72.6/113.3	40.7
	185.8/308.1	122.3
	324.6/551.7	227.2
	559.8/869.4	309.6
	870.3/1150.7	280.4
**5**-Tb(b)	0/3.9	3.9
	7.1/8.6	1.5
	73.2/91.9	18.7
	113.3/115.7	2.4
	115.8/168.1	52.4
	174.8/183.4	8.7
